# Resolution of sequence divergence for repeat-mediated deletions shows a polarity that is mediated by MLH1

**DOI:** 10.1093/nar/gkac1240

**Published:** 2023-01-09

**Authors:** Hannah Trost, Arianna Merkell, Felicia Wednesday Lopezcolorado, Jeremy M Stark

**Affiliations:** Department of Cancer Genetics and Epigenetics, Beckman Research Institute of the City of Hope, Duarte, CA 91010, USA; Irell and Manella Graduate School of Biological Sciences, Beckman Research Institute of the City of Hope, Duarte, CA 91010, USA; Department of Cancer Genetics and Epigenetics, Beckman Research Institute of the City of Hope, Duarte, CA 91010, USA; Department of Cancer Genetics and Epigenetics, Beckman Research Institute of the City of Hope, Duarte, CA 91010, USA; Department of Cancer Genetics and Epigenetics, Beckman Research Institute of the City of Hope, Duarte, CA 91010, USA; Irell and Manella Graduate School of Biological Sciences, Beckman Research Institute of the City of Hope, Duarte, CA 91010, USA

## Abstract

Repeat-mediated deletions (RMDs) are a type of chromosomal rearrangement between two homologous sequences that causes loss of the sequence between the repeats, along with one of the repeats. Sequence divergence between repeats suppresses RMDs; the mechanisms of such suppression and of resolution of the sequence divergence remains poorly understood. We identified RMD regulators using a set of reporter assays in mouse cells that test two key parameters: repeat sequence divergence and the distances between one repeat and the initiating chromosomal break. We found that the mismatch repair factor MLH1 suppresses RMDs with sequence divergence in the same pathway as MSH2 and MSH6, and which is dependent on residues in MLH1 and its binding partner PMS2 that are important for nuclease activity. Additionally, we found that the resolution of sequence divergence in the RMD product has a specific polarity, where divergent bases that are proximal to the chromosomal break end are preferentially removed. Moreover, we found that the domain of MLH1 that forms part of the MLH1-PMS2 endonuclease is important for polarity of resolution of sequence divergence. We also identified distinctions between MLH1 versus TOP3α in regulation of RMDs. We suggest that MLH1 suppresses RMDs with sequence divergence, while also promoting directional resolution of sequence divergence in the RMD product.

## INTRODUCTION

Repeat-mediated deletions (RMDs) are a type of chromosomal rearrangement involving recombination between two repeat elements that causes a deletion between the repeats, along with one of the repeats (1,2). A likely mechanism of RMDs is single-strand annealing (SSA), which involves a chromosomal break between two repeat elements that is resected to generate 3’ ssDNA that enables the two repeat elements to anneal together to bridge the DSB. Subsequent removal of 3’ non-homologous tails, fill-in synthesis, and ligation completes these events (Supplemental Figure S1A) (3). RMDs have the potential to reshape mammalian genomes, due to the high density of repetitive DNA elements, such as long interspersed elements and short interspersed elements, including approximately one million Alu-like elements in the human genome (4–7). Indeed, RMDs have been associated with several genetic diseases, including loss of tumor suppressor genes leading to increased cancer incidence (8,9). Notably, repeat elements show substantial sequence divergence, which is a potent suppressor of recombination between repeat sequences (10,11). For example, Alu-like elements can show up to 20% sequence divergence between elements (4).

Suppression of recombination between divergent sequences involves components of the mismatch repair pathway (12–14), which is a critical aspect of DNA replication to excise misincorporated bases (15–18). Mismatch repair begins with the mispair recognition complex of MSH2 and one of its two binding partners, MSH3 and MSH6 (18–23). The MSH2-MSH6 complex preferentially recognizes single-base mismatches and small insertion/deletion mispairs, whereas the MSH2-MSH3 complex recognizes relatively larger insertion/deletion mispairs (18–23). Upon mispair recognition, MSH2-MSH6 or MSH2-MSH3 recruits MLH1 and one if its binding partners: mammalian PMS2 (*Saccharomyces cerevisiae PMS1*), PMS1 (*S. cerevisiae MLH2*), or MLH3 (15–18,24–35). Each of these MLH1 binding partners has distinct properties. MLH1-MLH3 has nuclease activity, is involved in meiotic recombination, and may also play a specific role downstream of the MSH2-MSH3 heterodimer to repair specific insertion/deletion loops (25–29). MLH1-PMS1 (*S. cerevisiae MLH2*) lacks nuclease activity, but is recruited to sites of mismatches in a manner dependent on MSH2, suppresses the frequency of frameshift mutations, and suppresses mutation rates when combined with reduced expression of PMS2 (*S. cerevisiae PMS1*) (30,31). Finally, MLH1-PMS2 has nuclease activity and creates a nick upstream of the site of the mismatch, which initiates excision of the nicked strand (32–35). Displacement of the nicked strand could occur by excision via the exonuclease EXO1 or EXO1-independent pathways, which include a process mediated by RAD27/FEN1, iterative nicking via MLH1-PMS2, and/or displacement synthesis (36–39).

There are apparent mechanistic distinctions between mismatch repair during DNA replication versus suppression of recombination between divergent sequences. For example in *S. cerevisiae*, both MSH6 and MLH1 are required for mismatch repair; but only MSH6 is required for suppression of DSB-induced SSA events between divergent sequences, whereas MLH1 appears dispensable (40–42). Similarly, for an ectopic mitotic DSB-induced recombination assay between divergent sequences in *S. cerevisiae*, only MSH6, but not MLH1, suppress crossover recombination, whereas both factors suppress non-crossover recombination events (43). In contrast, for spontaneous recombination events between divergent sequences, MLH1-PMS2 appears to suppress these events, albeit often to a lesser degree than MSH2. For one, MLH1-PMS2 (PMS1 in *S. cerevisiae*) appears to suppress spontaneous homologous recombination between divergent inverted repeats that involves PMS2 nuclease function, along with requiring MSH2 (44–46). However, with such inverted repeat recombination, in cells lacking the RAD51 recombinase, loss of MSH2 and MLH1 caused similar effects, whereas in cells lacking RAD59 (a paralog of the recombination mediator RAD52), loss of MSH2 showed a greater effect versus MLH1 (47). Similarly, both PMS2 (PMS1 in *S. cerevisiae*) and MSH2 were shown to suppress spontaneous mitotic crossover recombination between divergent sequences, but the influence of MSH2 was much greater (48,49). Also consistent with this pattern, studies of spontaneous gross chromosomal rearrangements (GCRs) in *S. cerevisiae* revealed that MSH2 and MLH1 appear to specifically suppress duplication-mediated versus single-copy sequence mediated GCRs, but again, MSH2 showed a greater effect versus MLH1 (50). Thus, the specific circumstances appear to affect the relative requirements for MSH2 versus MLH1 for suppressing recombination between divergent sequences in *S. cerevisiae*.

Whether such mechanistic distinctions between mismatch repair and regulation of homologous recombination are conserved in mammalian cells has been unclear, as are other aspects of the role of mismatch repair in regulation of RMDs in mammalian cells. For example, the mechanisms and patterns of resolution of divergent sequences during RMDs have been poorly understood. Additionally, the relationship between mismatch repair and other factors important for suppression of recombination between divergent sequences is unclear. In this study, we have used an assay system for RMDs in mouse cells to survey the influence of several DNA damage response factors on distinct RMD events, and subsequently focus on defining the role of MLH1 on regulation of RMDs between divergent repeats, including for resolution of sequence divergence.

## MATERIALS AND METHODS

### Oligonucleotides, plasmids and cell lines

The siRNAs were pools of 4 per gene in equal concentrations, which were from Dharmacon, with the catalog numbers and sequences in Supplemental Table S1. The non-targeting siRNA (siCTRL) was Dharmacon #D001810-01 5'-UGGUUUACAUGUCGACUAA. Other oligonucleotides are in Supplemental Table S2. The reporter plasmids RMD-GFP, 1%RMD-GFP, 3%RMD-GFP were previously described (1). All sgRNA/Cas9 plasmids used the px330 plasmid (Addgene 42230, deposited by Dr. Feng Zhang) (51). The sgRNA sequences for inducing DSBs in the reporters were previously described (1), apart from the 1 kb DSB, which is in Supplemental Table S2. The plasmids pCAGGS-NZE-GFP (GFP expression vector), pgk-puro, and pCAGGS-BSKX empty vector (EV) were described previously (52). The expression vectors for MLH1, TOP3α, and PMS2 were generated with gBLOCK (Integrated DNA Technologies) insertions into pCAGGS-BSKX, with the latter two including silent mutations to mutate all four siRNA target sequences. The mutant forms of TOP3α (Y362F) and PMS2 (E702K) were also generated with gBLOCKs, whereas for the MLH1 mutant (Δ754–756) PCR was used to create a fragment with this deletion.

Several mESC lines with the RMD reporters were previously described: WT (1), *Msh2^−^^/^^−^* (1,53), and *Exo1^−^^/^^−^* (54,55). The *Mlh1^−^^/^^−^* mESC line was derived using two Cas9-mediated DSBs to introduce a deletion in *Mlh1* using the following sgRNAs, cloned into px330: 5’ CATTGACGTCCACGTTCTGA and 5’ CGAAGTTCACTTTCTGCACG. WT mESCs were transfected with these plasmids and pgk-puro using Lipofectamine 2000 (Thermofisher), transfected cells were enriched using transient puromycin (Sigma Adrich) treatment, followed by plating at low density to isolate and screen individual clones for loss of MLH1. The reporter plasmids were integrated into the *Pim1* locus of mESCs using electroporation of linearized plasmid, selection in hygromycin, and screening by PCR, as described previously (56).

### DSB reporter assays

For the RMD assays including siRNA, mESCs were seeded on a mixture of 3.75 pmol of each siRNA pool using RNAiMAX (Thermofisher) at a cell density of 0.5  ×  10^5^ cells per well of a 24-well plate, with 0.5 ml of antibiotic-free media. The next day, each well was transfected with 200 ng of each sgRNA/Cas9 plasmid plus 3.75 pmol of each siRNA pool using Lipofectamine 2000 (Thermofisher), with 0.5 ml of antibiotic-free media. For the RMD assays with expression vectors for various genes, transfections included 200 ng of these vectors, or the EV control (pCAGGS-BSKX). For the EJ7-GFP assay for NHEJ (No Indel EJ), cells were seeded in the same conditions as the RMD reporters, using the two sgRNAs for this assay, as described (57). For all reporter assays, three days after transfection, cells were analyzed by flow cytometry using a CyAn-ADP or ACEA Quanteon, as described (52).

Each experiment included parallel transfections with the GFP expression vector, along with the respective expression vectors and/or siRNAs, to normalize all repair frequencies to transfection efficiency. Namely, each GFP + frequency for an RMD event for a given condition is divided by the GFP+ frequency for the corresponding parallel transfections for that condition using the GFP expression vector. Some siRNA experiments are also normalized to non-targeting siRNA (siCTRL). For this normalization, the GFP+ frequency normalized to transfection efficiency is divided by the mean value of the parallel siCTRL transfections, such that the mean siCTRL value is 1 (i.e. siCTRL = 1).

### Resolution of sequence divergence in final RMD products analysis

For the resolution of divergent sequences in final RMD products with 3%RMD-GFP, the transfection conditions were the same as the frequency analysis described above, and all included siRNA (either siCTRL or siTOP3α), except all amounts were scaled at 2-fold to a 12-well dish, and three days after transfection cells were expanded prior to sorting for GFP + cells, which were cultured for sorting a second time (BD Aria). Genomic DNA from these samples, purified by phenol/chloroform extraction as described (52), was used to amplify the repeat sequence using RMDjunct368UPillumina and RMDjunct368DNillumina primers, which include the Illumina adapter sequences. The amplicons were subjected to deep sequencing using the Amplicon-EZ service (AZENTA/GENEWIZ), which includes their SNP/INDEL detection pipeline, which aligned the reads to the top strand sequence (Supplemental Figure S1B) as the reference sequence. All reads that represented ≥ 0.1% of the total reads for each sample were individually aligned to the reference sequence, and each of the 8 the mismatches were identified as being from either the top or bottom strand (Supplemental Figure S1B), which was used to calculate the percentage of top strand base retention for each mismatch location. Each cellular condition was examined with three independent transfections and GFP + sorted samples, and the percentage of retention of the top strand base from the three samples was used to calculate the mean and standard deviation.

### Immunoblotting and quantitative reverse transcription PCR (qRT-PCR).

For immunoblotting analysis, cells were transfected using the same total siRNA and plasmid concentrations as for the reporter assays, but using EV instead of sgRNA/Cas9 plasmids and scaled 2-fold using a 12-well dish. For analysis of siRNA treated cells, following the pre-treatment with siRNA using RNAiMAX (Thermofisher), cells were transfected with pgk-puro plasmid (400 ng), EV (800 ng), and siRNA (7.5 pmol each siRNA pool). The next day, cells were re-plated into puromycin and cultured for two days to enrich for transfected cells. Cells were lysed with ELB buffer (250 mM NaCl, 5 mM EDTA, 50 mM Hepes, 0.1% (v/v) Ipegal, and Roche protease inhibitor) with sonication (Qsonica, Q800R). Blots were probed with antibodies for CtIP (Active Motif 61141), BLM (Bethyl Laboratories A300-110A), MLH1 (Abcam, ab92312), MSH6 (Proteintech, 18120–1AP), MSH2 (Bethyl Laboratories, A300-452), EXO1 (Bethyl Laboratories, A302-640A), TOP3α (Proteintech, 14525I-AP), FLAG (Sigma, A8592) and ACTIN (Sigma, A2066). Secondary antibodies (Abcam, ab205719, ab205718). ECL reagent (Amersham Biosciences) was used to develop immunoblotting signals. For quantitative RT-PCR (qRT-PCR) analysis to examine mRNA levels, total RNA was isolated (Qiagen RNAeasy), and reverse transcribed with MMLV-RT (Promega). The RT reactions were amplified with primers for target RNA and ACTIN (Supplemental Table S2) using iTaq Universal SYBR Green (Biorad, 1725120), and quantified on (Biorad CRX Connect Real-Time PCR Detection System, 1855201). Relative levels of each mRNA were determined using the cycle threshold (Ct) value for target mRNA for individual PCR reactions subtracted by Ct value for the ACTIN control (ΔCt value), which was then subtracted from the corresponding ΔCt from siCTRL treated cells (ΔΔCt), which was then used to calculate the 2^−ΔΔCt^ value.

### Tracking of indels by DEcomposition (TIDE) analysis

WT mESCs were transfected using the same total plasmid concentrations as for the reporter assays, but using sgRNA/Cas9 plasmids and pgk-puro plasmid, and scaled 2-fold using a 12-well dish. The next day, cells were re-plated into puromycin and cultured for two days to enrich for transfected cells. Subsequently, genomic DNA samples were amplified using primers flanking the predicted DSB location, the PCR products were gel purified and analyzed by Sanger sequencing (City of Hope Integrative Genomics Core, Applied Biosystems 3730 DNA Analyzer), which was used for TIDE analysis (58) to determine the frequency of indels (% INDEL).

## RESULTS

### Components of mismatch repair and the BLM-TOP3α-RMI1/2 (BTR) complex suppress RMDs, whereas several other factors promote these events

**Figure 1.**
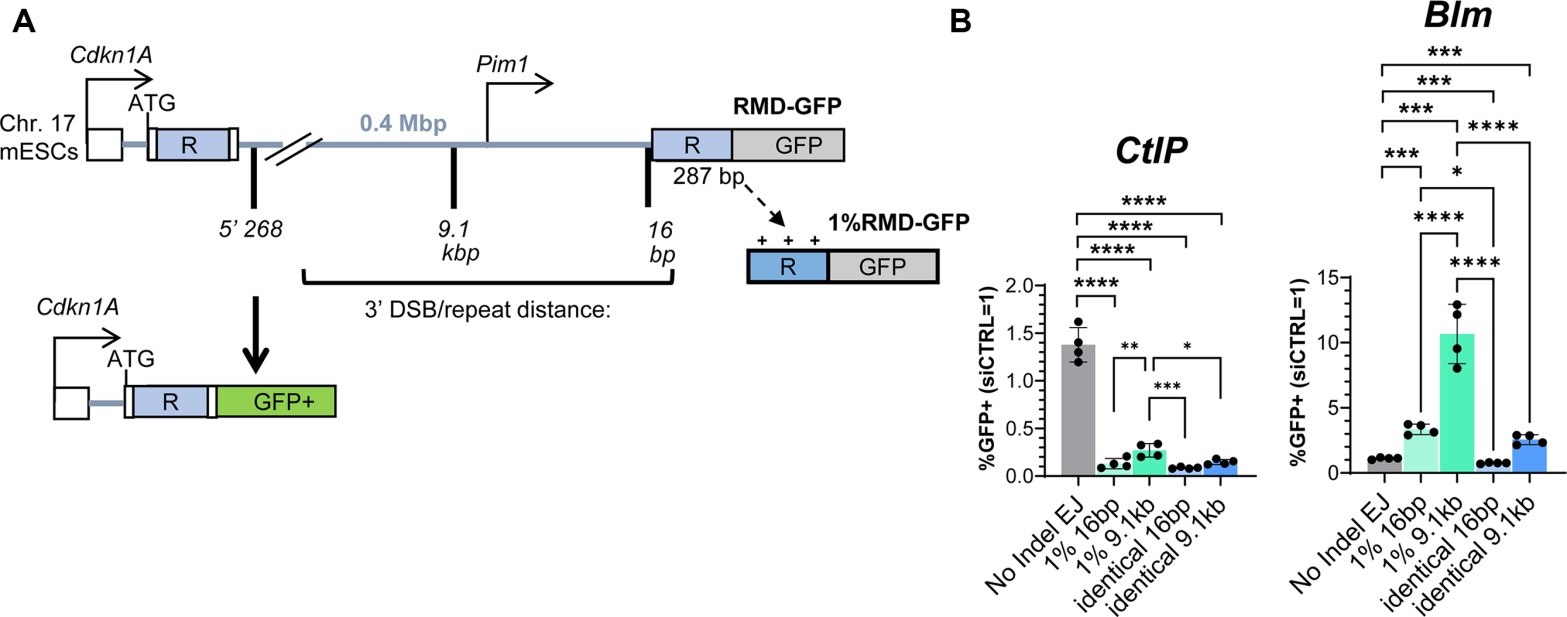
Assay system for examining RMDs that are affected by CtIP and BLM. (**A**) Shown is the RMD-GFP reporter, which is integrated into the *Pim1* locus in chromosome 17 of mESCs, such that repair of two DSBs by an RMD leads to GFP + cells. The two repeats shown as ‘R’, the 5’ repeat being endogenous sequence and the 3’ repeat is fused to GFP. 1%RMD-GFP has 1% sequence divergence between the repeats. RMDs are induced by creating two DSBs: one 268 bp downstream of the 5’ repeat, and the other either 16 bp or 9.1 kb upstream of the 3’ repeat, which we refer to as the DSB / repeat distance. (**B**) Shown are the effects of siRNAs targeting BLM (siBlm) and CtIP (siCtIP) for 4 RMD reporter assays, and an NHEJ assay (EJ7-GFP, No Indel EJ). Repair frequencies are normalized to transfection efficiency, and parallel non-targeting siRNA (siCTRL = 1). *n* = 4. *P ≤ 0.05, **P ≤ 0.005, ***P ≤ 0.0005, ****P < 0.0001, Statistics are one-way ANOVA using Tukey's multiple comparisons test.

We sought to identify DNA damage response factors that influence the formation of RMDs, using a reporter system that uses GFP expression as a measure of RMDs, called RMD-GFP (Figure 1A) (1). This reporter has two tandem 287 bp repeats (shown as ‘R’) separated by 0.4 Mbp on chromosome 17 in mouse embryonic stem cells (mESCs). The 5’ repeat is the endogenous sequence located just downstream of the *Cdkn1A* promoter, and the 3’ repeat is targeted to the *Pim1* locus and is fused to GFP. An RMD between these two repeats generates a *Cdkn1A-GFP* fusion gene that causes GFP + cells, which can be measured with flow cytometry. To induce an RMD, we introduce two DSBs between the two repeats using Cas9/sgRNAs. The 5’ DSB is always at the same position, which is 268 bp downstream of the 5’ repeat (5’ 268 bp). The 3’ DSB can be made at various distances upstream of the 3’ repeat, which we refer to as the DSB/repeat distance. There are also two other versions of the RMD-GFP reporter that contain equally spaced mismatches in the 3’ repeat: 1%RMD-GFP with three mismatches causing 1% sequence divergence, and 3%RMD-GFP with eight mismatches causing 3% sequence divergence (Supplemental Figure S1B). All assay conditions are normalized to transfection efficiency with parallel transfections with a GFP expression vector.

To begin with, we examined two factors already implicated in RMD regulation (1,55), which served as controls during our survey of other factors, as described below. Specifically, we examined effects of siRNA knockdown of the BLM helicase and the end resection factor CtIP on four versions of the RMD-GFP assay: (i) RMD-GFP with the 16 bp DSB/repeat distance, (ii) RMD-GFP with the 9.1 kb DSB/repeat distance, (iii) 1% RMD-GFP with the 16 bp DSB/repeat distance and (iv) 1% RMD-GFP with the 9.1 kb DSB/repeat distance. We chose these versions of the assay as it enables a comparison of identical versus divergent repeats, each at both very short and relatively long DSB/repeat distances. We also included an assay for non-homologous end joining (NHEJ) as a control (EJ7-GFP/No Indel EJ assay; Supplemental Figure S1C) (57). This NHEJ assay involves a GFP cassette interrupted by a spacer sequence and use of two sgRNAs that target Cas9 to induce blunt DSBs to precisely excise this spacer sequence. Subsequent repair of the distal blunt DSB ends without insertion/deletion mutations restores the GFP+ cassette, which is dependent on several NHEJ factors (e.g. XRCC4) (57).

With such analysis of four RMD events and NHEJ, we found that depleting the end resection factor CtIP causes a significant decrease in all four RMDs compared to NHEJ, although the 1% RMD-GFP (i.e. divergent repeat) assay with the 9.1 kb DSB/repeat distance was affected the least (Figure 1B). In contrast, BLM knockdown caused a specific increase in three of the RMDs (i.e. RMDs with both identical and divergent repeats at the 9.1 kb DSB/repeat distance, and the divergent repeat at 16 bp), and a modest decrease for the identical repeat at 16 bp, each compared to the effect on NHEJ (Figure 1B). Also, the fold-effects of BLM knockdown differed among the RMD events, with the divergent repeat at 9.1 kb showing a markedly greater effect (Figure 1B). We confirmed siRNA knockdown of CtIP and BLM with both qRT-PCR and immunoblotting (Supplemental Figure S2A, B).

**Figure 2.**
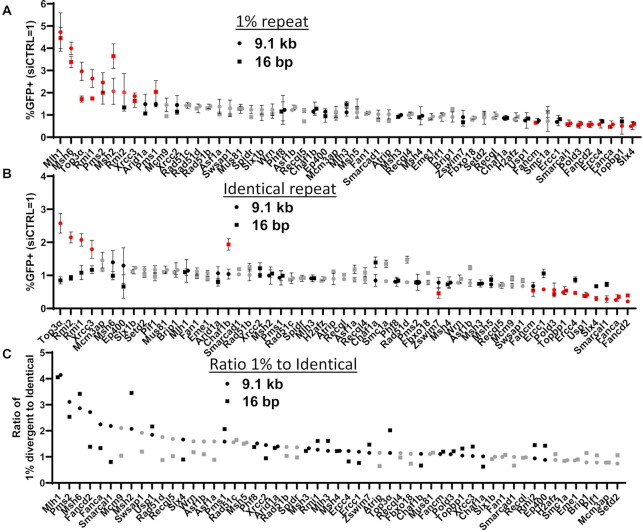
Components of mismatch repair and the BLM-TOP3α-RMI1/2 (BTR) complex suppress RMDs, whereas several other factors promote these events. (**A**) Shown are effects of siRNAs targeting 55 factors on frequency of RMDs at both 16 bp and 9.1 kb DSB/repeat distances for 1%RMD-GFP. Frequencies are normalized to transfection efficiency and parallel siCTRL (= 1). Genes are ranked by the fold-effect relative to siCTRL at 9.1 kb. All siRNAs tested *n* = 2, and those with ≥1.5-fold effect from these trials were tested a total of *n* = 4. Grey: *n* = 2, black: *n* = 4, red: *n* = 4 and also ≥1.5-fold effect relative to siCTRL. (**B**) Shown are the effects of the siRNAs targeting 55 factors on frequency of RMDs at both 16 bp and 9.1 kb DSB/repeat distances for RMD-GFP. Colors and analysis as in (A). (**C**) Ratio of divergent versus identical RMD frequencies. For the data shown in (A) and (B), shown is the ratio of RMD frequencies from 1% RMD-GFP divided by RMD-GFP. Grey: *n* = 2, black: *n* = 4. As above, genes are ranked by the fold-effect relative to siCTRL at 9.1 kb. Data are represented as mean values ± SD.

**Figure 3.**
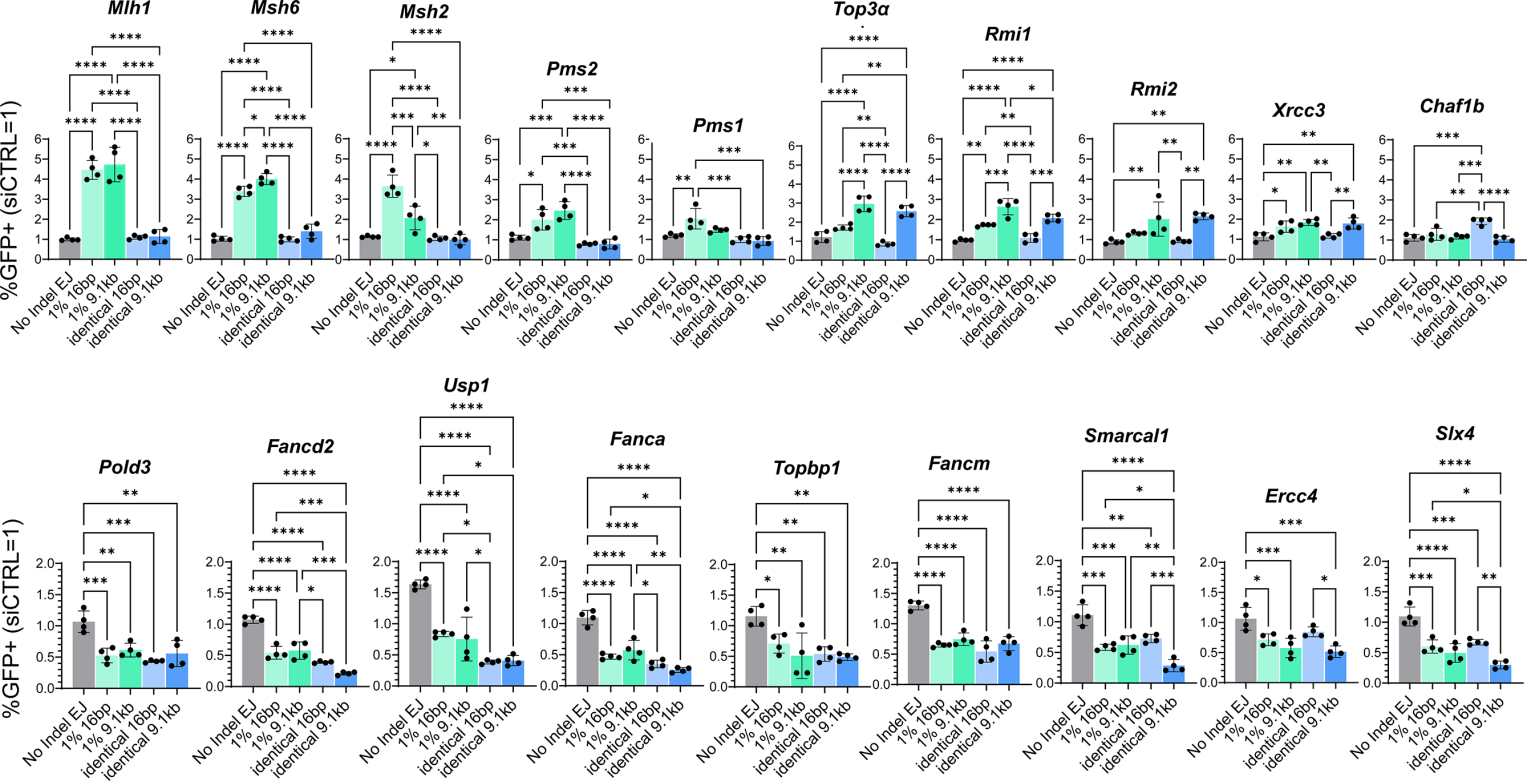
A set of siRNAs targeting 19 different DNA damage response factors caused a significant difference in at least one RMD event relative to NHEJ. Shown are effects of siRNAs against 19 genes on four RMD events, and NHEJ (No Indel EJ). Frequencies are normalized to transfection efficiency and parallel siCTRL (= 1). *n* = 4. *P ≤ 0.05, **P ≤ 0.005, ***P ≤ 0.0005, ****P < 0.0001, one-way ANOVA using Tukey's multiple comparisons test. Data are represented as mean values ± SD.

Using these four variants of the RMD assay, we then sought to identify other factors involved in RMD regulation by surveying effects of siRNAs targeting 55 factors involved in chromatin and the DNA damage response, mismatch repair, and DNA annealing and/or end processing. We measured the effects of siRNAs (pool of 4 per gene) against 55 targets on the frequency of the four RMD events described above, which were compared parallel treatments with a non-targeting siRNA (siCTRL). Each siRNA was tested on all four RMD assays in duplicate, and repeated if the initial fold-effect for any of the assays was ≥1.5-fold. We then ranked the results based on the normalized fold-effect at 9.1 kb for both 1%RMD-GFP and RMD-GFP (Figure 2A, B). For comparison, we also determined the ratio of divergent versus identical RMDs for each siRNA (i.e. frequencies of 1%RMD-GFP divided by RMD-GFP) (Figure 2C). From the analysis of individual RMD events, we found siRNAs targeting 22 factors caused a ≥1.5-fold effect on at least one of the four RMD assays (Figure 2A, B, highlighted in red). We then examined these 22 factors using the NHEJ assay, and performed a one-way ANOVA with a Tukey's post-test to compare the fold-effects between all five assays: the four RMD events and NHEJ. We found that siRNAs targeting 19 of the 22 factors caused a significant difference in at least one RMD event relative to NHEJ, and for all 22 siRNAs we confirmed knockdown of the target RNA via qRT-PCR (Figure 3, Supplementary Figure S3A, B). Additionally, we were able to confirm knockdown of the target RNA via qRT-PCR for 29 of the other 33 factors that failed to cause a ≥1.5-fold effect on at least one of the four RMD assays (all targets except Rad51b, Rad51c, Recql4, Recql5, Supplemental Figure S4).

The 19 factors fell into different categories based on the relative effects on the distinct RMDs (Figure 3). Nine of the factors, several of which are in the Fanconi Anemia pathway, (POLD3, FANCD2, USP1, FANCA, TOPBP1, FANCM, SMARCAL1, ERCC4 and SLX4) had similar effects as CtIP. Namely, knockdown of these factors caused a significant decrease in all four RMD events. Indeed, for the RMD between identical repeats, using the 9.1 kb DSB/repeat distance, siRNAs targeting four factors (FANCD2, FANCA, SMARCAL1 and SLX4) caused a substantial decrease (i.e. >3-fold). In contrast, siRNAs targeting the remaining ten factors (MLH1, MSH6, MSH2, PMS2, PMS1, TOP3α, RMI1, RMI2, XRCC3 and CHAF1B) caused an increase in at least one RMD event, indicating these factors suppress RMDs. The siRNAs targeting MLH1, MSH6, MSH2 and PMS2 each caused a significant increase in RMDs with repeat divergence irrespective of DSB/repeat distance, and had no effect on RMDs with identical repeats. Similarly, siRNAs targeting PMS1 caused an increase in RMDs with repeat divergence (1%RMD-GFP) but only for the 16 bp DSB/repeat distance. Thus, these factors (MLH1, MSH6, MSH2, PMS2 and PMS1) appear to suppress RMDs with repeat divergence. The pattern is more complex with siRNAs targeting TOP3α, RMI1 and RMI2, which are components of the BTR complex (BLM-TOP3α-RMI1/2). Specifically, these factors caused the greatest fold-increases for the 9.1 kb DSB/repeat distance irrespective of repeat divergence, followed by a more modest increase for 16 bp with 1% RMD-GFP, and no statistical difference for 16 bp with RMD-GFP (Figure 3). Finally, the siRNA targeting CHAF1B caused a specific increase with RMD-GFP at the 16 bp DSB/repeat distance, and conversely targeting XRCC3 caused a modest increase in each of the RMD events except with RMD-GFP at the 16 bp DSB/repeat distance. Altogether, these findings indicate that factors from several pathways, including the Fanconi Anemia pathway, mismatch repair, and the BTR complex influence RMD formation in ways that can be affected by repeat divergence and/or DSB/repeat distance.

### MLH1 suppresses RMDs with divergent repeats

**Figure 4.**
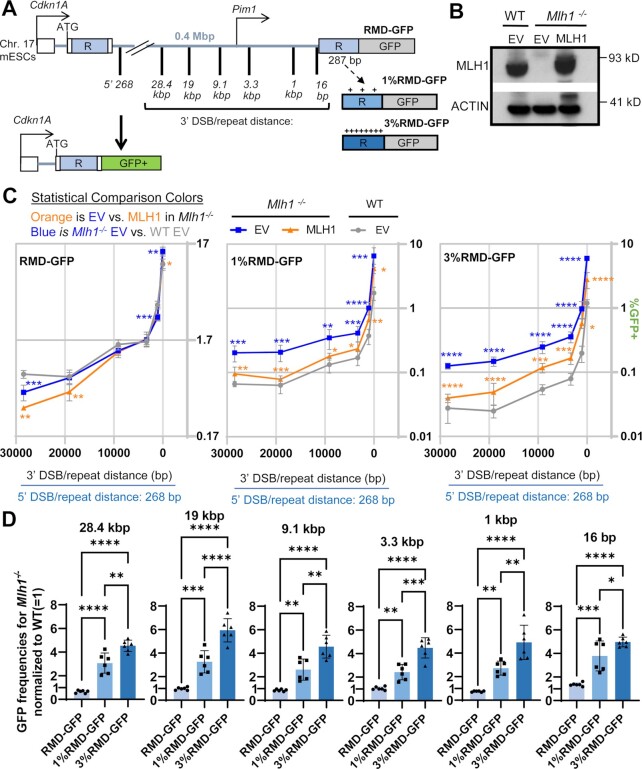
MLH1 suppresses RMDs with divergent repeats. (**A**) Shown is the RMD-GFP reporter as in Figure 1A, but with all DSB sites represented, and with the three repeat sequence versions: identical, 1% RMD-GFP (1% divergence) and 3% RMD-GFP (3% divergence). (**B**) Immunoblotting analysis of MLH1 and ACTIN in WT and *Mlh1^−^^/^^−^* mESCs transfected with either EV or MLH1 vectors. (**C**) RMD frequencies for the assays shown in (A), normalized to transfection efficiency, for WT transfected with empty vector (EV), *Mlh1^−^^/−^*transfected with EV, and *Mlh1^−^^/^^−^* transfected with MLH1 expression vector. *n* = 6. *P ≤ 0.05, **P ≤ 0.005, ***P ≤ 0.0005, ****P < 0.0001, unpaired *t*-test with Holm−Sidak correction. Asterisk colors represent specific comparisons, as shown. (**D**) Effects of sequence divergence on the relative influence on MLH1 on RMDs. RMD frequencies *Mlh1^−^^/^^−^* shown in (C) were normalized to WT (= 1), and grouped by location of the DSB upstream of the 3’ repeat to enable comparisons of effects of sequence divergence. *n* = 6. *P ≤ 0.05, **P ≤ 0.005, ***P ≤ 0.0005, ****P < 0.0001, one-way ANOVA with Tukey's multiple comparisons test. Data are represented as mean values ± SD.

Based on the above survey, we chose to focus on MLH1, both because of its marked effect on the RMDs with divergent repeats, and because its influence on regulation of RMDs, and indeed homologous recombination in mitotic mammalian cells, remains poorly understood. We first generated an *Mlh1^−^^/^^−^* mESC line by targeting sgRNAs/Cas9 to exon 11 of *Mlh1* that we confirmed has loss of MLH1 by immunoblotting (Figure 4B). We also created an MLH1 expression vector that we validated with immunoblotting (Figure 4B). We then integrated the three RMD reporters (RMD-GFP, 1% RMD-GFP, 3% RMD-GFP) in the *Mlh1^−^^/−^* mESC line, and these RMD assays were tested using six different 3’ DSB/repeat distances: five that were previously described (16 bp, 3.3, 9.1, 19, 28.4 kb) (1), whereas the sixth (1 kb) was added for this study to fill a gap between 16 bp and 3.3 kb. To validate the 1 kb DSB site, we used TIDE (tracking of indels by decomposition) analysis (58), which confirmed induction of indels at the predicted 1 kb DSB site (Supplemental Figure S5). Also with this TIDE analysis, we found that indel frequencies for the 1 kb DSB site were similar to the 16 bp and 9.1 kb DSB sites (Supplemental Figure S5). We compared the results of the RMD assays in the *Mlh1^−^^/^^−^* cell lines to WT cells (transfected with empty vector, EV), and also to the complemented condition (*Mlh1^−^^/-^*transfected with the MLH1 complementation vector) (Figure 4C).

From this analysis, MLH1 showed largely no effect on RMDs between identical repeats, although mild (≤1.5-fold) effects were observed at 28.4 kb, 1 kb and 16 bp (*Mlh1^−^^/^^−^* versus WT, Figure 4C). However, for RMDs with divergent repeats (1% and 3%), loss of MLH1 caused a significant increase in RMDs at all DSB/repeat distances, both by comparing *Mlh1^−^^/^^−^* versus WT, and versus the complemented cells (*Mlh1^−^^/^^−^* cells transfected with the MLH1 expression vector, Figure 4C). We then compared the fold effects of MLH1 loss (*Mlh1^−^^/^^−^* versus WT) among the degrees of repeat divergence (identical, 1% and 3%) for each DSB/repeat distance. Loss of MLH1 caused a significant increase in RMDs at all DSB/repeat distances in 1%RMD-GFP compared to RMD-GFP, and in 3%RMD-GFP compared to 1% RMD-GFP. Thus, the role of MLH1 in suppressing RMDs increased as divergence between the repeats increased (Figure 4D). In contrast, the role of MLH1 was not significantly different between distinct DSB/repeat distances for 1% RMD-GFP and 3% RMD-GFP, although some minor statistical differences based on DSB/repeat distance were observed for RMD-GFP (Supplemental Figure S6A). These findings indicate that MLH1 is critical to suppress RMDs if the repeats contain sequence divergence, irrespective of DSB/repeat distance.

### MLH1, MSH2, and MSH6 function in the same pathway, but independently of EXO1, for suppression of RMDs

Because MLH1 is part of the mismatch repair pathway, we compared its effect to other mismatch repair components and also tested effects of combined mutants of mismatch repair factors. During mismatch repair, the MSH2 and MSH6 complex recognizes sites of mismatches to then recruit MLH1-PMS2 for strand nicking (15). Excision of the nicked strand occurs both by EXO1-dependent and EXO1-independent pathways, which include excision via RAD27/FEN1, and iterative nicking via MLH1-PMS2 (36–39). To examine the interplay between these factors for RMD regulation, we examined effects of depleting MLH1 and MSH6 in WT, *Mlh1^−^^/^^−^*, *Msh2^−^^/^^−^* and *Exo1^−^^/−^* mESCs. For this analysis, we tested all three RMD reporters (identical repeats, 1% and 3% divergent repeats), each at the 16 bp and 9.1 kb DSB/repeat distances.

**Figure 5.**
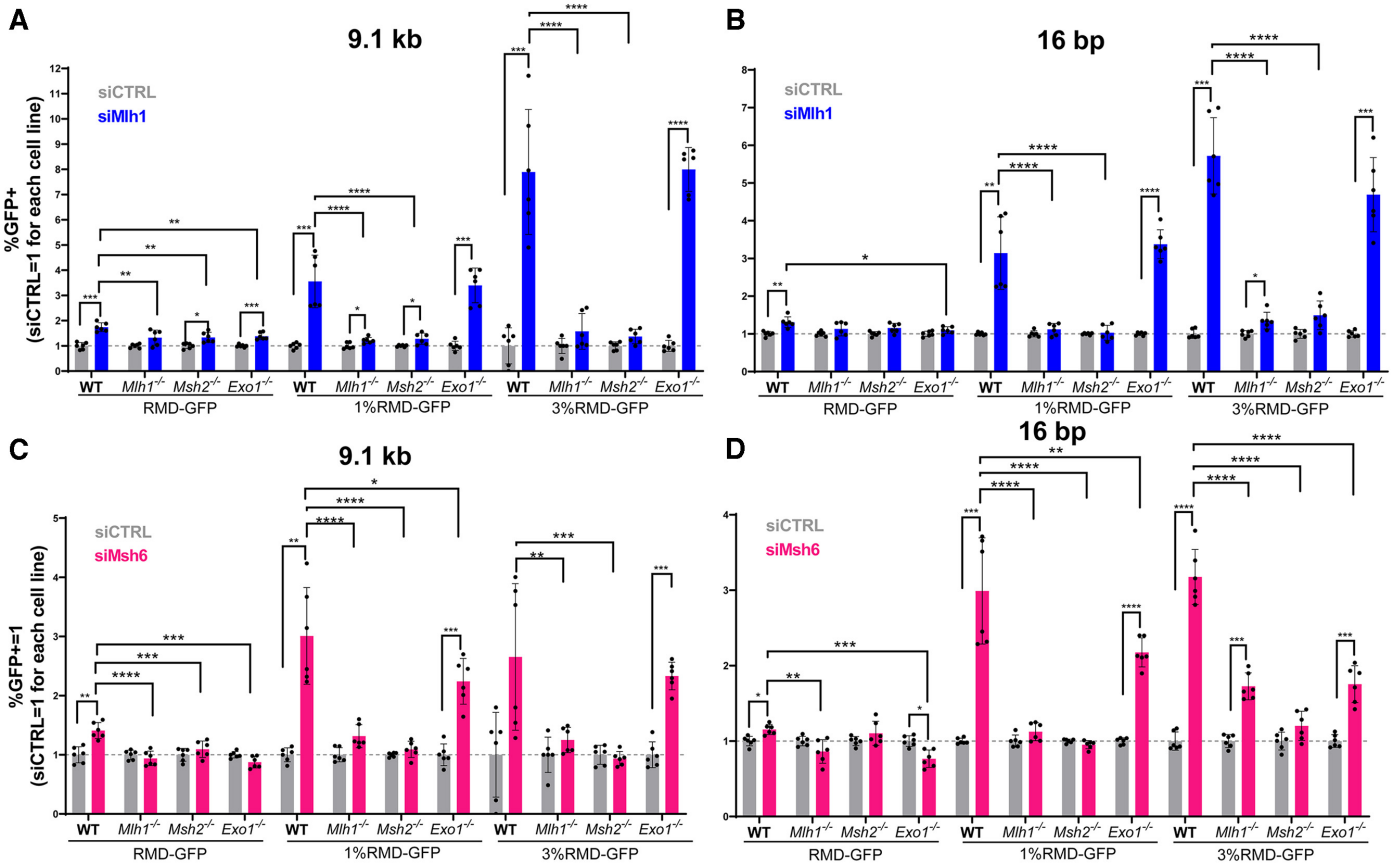
MLH1, MSH2 and MSH6 function in the same pathway, but function independently of EXO1, for suppression of RMDs. (**A**) Shown is the effect of siRNAs targeting MLH1 (siMlh1) on three RMD events (9.1 kb DSB/repeat distance, RMD-GFP, 1% GFP-GFP, 3% RMD-GFP) in WT, *Mlh1^−^^/^^−^*, *Msh2^−^^/^^−^* and *Exo1^−^^/^^−^* mESCs. Frequencies are normalized to transfection efficiency and parallel siCTRL (= 1). *n* = 6. *P ≤ 0.05, **P ≤ 0.005, ***P ≤ 0.0005, ****P < 0.0001, unpaired *t*-test for siCTRL versus siMlh1, and unpaired *t*-test using Holm−Sidak correction for effect of siMlh1 in WT versus the other genetic backgrounds. (**B**) Shown is the analysis as in (A), but using the 16 bp DSB/repeat distance. *n* = 6. Statistics as in (A). (**C**) Shown is the analysis as in (A), but for effects of siRNAs targeting MSH6 (siMsh6). *n* = 6. *P ≤ 0.05, **P ≤ 0.005, ***P ≤ 0.0005, ****P < 0.0001, unpaired *t*-test for siCTRL versus siMsh6, and unpaired *t*-test using Holm−Sidak correction for effect of siMsh6 in WT versus the other genetic backgrounds. (**D**) Shown is the analysis in (C), but using the 16 bp DSB/repeat distance *n* = 6. Statistics as in (C). Data are represented as mean values ± SD.

We found that knockdown of MLH1 in WT and *Exo1^−^^/^^−^* mESCs caused a marked increase in RMDs between the divergent repeats at both DSB/repeat distances, but not identical repeats (Figure 5A, B). In contrast, knockdown of MLH1 in *Msh2^−^^/^^−^* mESCs failed to cause an increase in any of the RMD events tested (Figure 5A, B). Although, for both WT and *Msh2^−^^/^^−^* mESCs, the Mlh1 siRNA did not cause complete knockdown, as measured by MLH1 immunoblot analysis (Supplemental Figure S6B). We found analogous results with MSH6, in that knockdown of this factor caused an increase in RMDs between divergent repeats in both WT and *Exo1^−^^/^^−^* mESCs, but not in *Mlh1^−^^/^^−^* and *Msh2^−^^/^^−^* mESCs (Figure 5C, D). We confirmed knockdown of MLH1 and MSH6 in each of the genetic backgrounds via immunoblotting (Supplemental Figure S6B, C). These results indicate that MLH1 and MSH6 suppress RMDs with divergent repeats independently of EXO1, but function in the same pathway as each other and MSH2.

### The MLH1-PMS2 endonuclease is important to suppress RMDs between divergent repeats

We next examined the mechanism by which MLH1 may suppress RMDs. MLH1 interacts with several proteins, including three heterodimer binding partners to form the MLH1-PMS2, MLH1-PMS1 and MLH1-MLH3 complexes (59). Furthermore, the MLH1-PMS2 and MLH1-MLH3 complexes have endonuclease activity (30,31,59). In our siRNA survey described above, we found that siRNAs targeting MLH3 did not affect RMDs, whereas siRNAs targeting PMS2 and PMS1 individually caused a ≥1.5-fold increase in RMDs with divergent repeats (Figure 2A). Thus, we sought to further evaluate the influence of MLH1-PMS2, MLH1-PMS1, as well as the role of the endonuclease domain of MLH1-PMS2 on RMDs.

**Figure 6.**
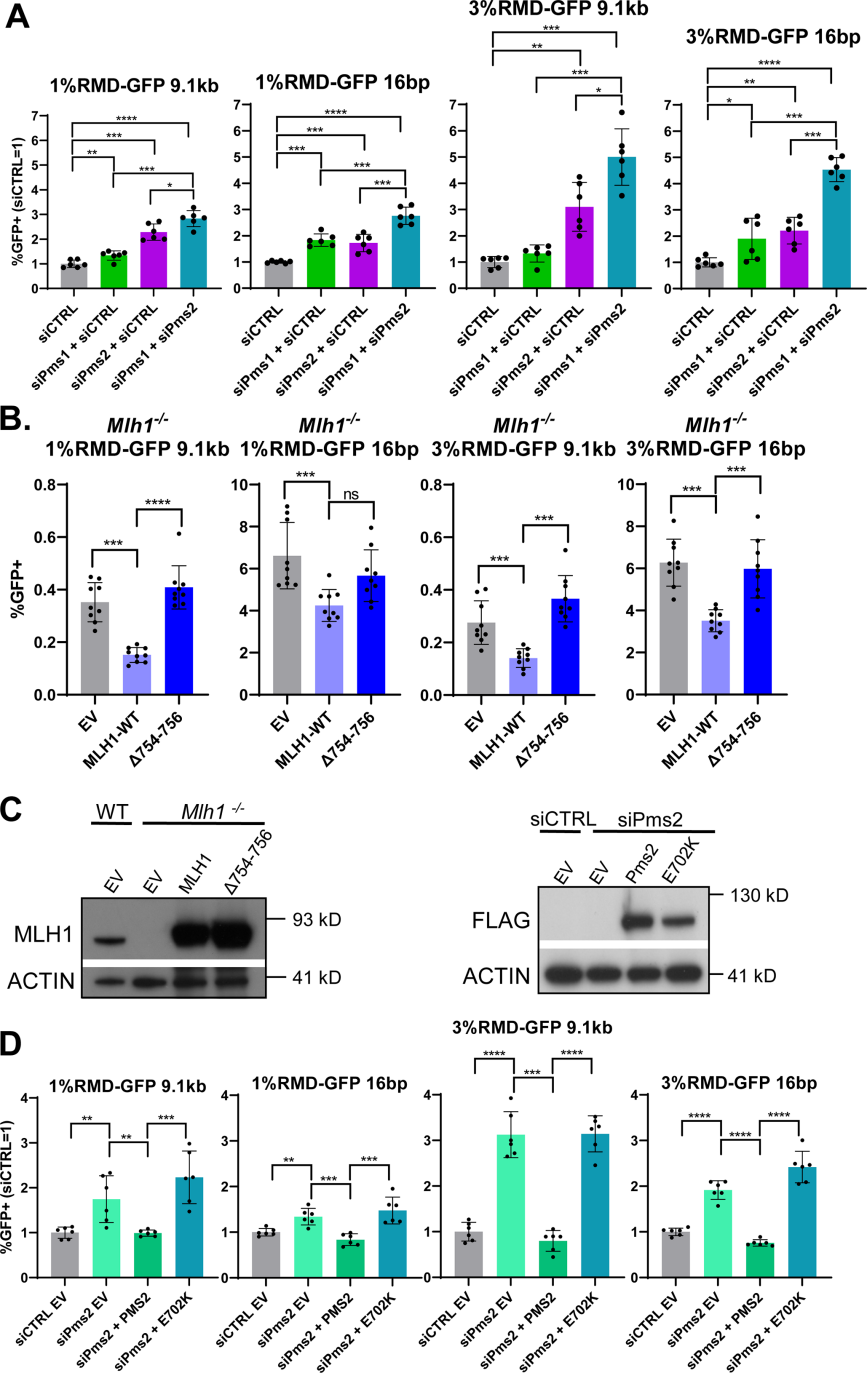
The MLH1-PMS2 endonuclease is important to suppress RMDs between divergent repeats. (**A**) Shown are the effects of siRNAs targeting PMS2 (siPms2) and Pms1 (siPms1) individually, and in combination (siPms2 + siPms1), on four RMD events: 1%RMD-GFP, 3%RMD-GFP, each at the 9.1 kb and 16 bp DSB/repeat distances. siCTRL is added to the siRNA treatments targeting the individual genes to ensure the same total siRNA concentration. Frequencies are normalized to transfection efficiency and parallel siCTRL (= 1). *n* = 6. *P ≤ 0.05, **P ≤ 0.005, ***P ≤ 0.0005, ****P < 0.0001, siCTRL versus each set of siRNA treatments, and also the combination (siPms2 + siPms1) versus the individual genes, each with unpaired *t*-test using Holm−Sidak correction. (**B**) Shown are RMD frequencies for the four RMDs shown in (A) in *Mlh1^−^^/^^−^* mESCs transfected with EV, MLH1-WT or MLH1-Δ746–756 that deletes the three residues at the C-terminus. Frequencies are normalized to transfection efficiency. *n* = 9. ***P ≤ 0.0005, ****P < 0.0001, ns = not significant, unpaired *t*-tests with Holm−Sidak correction. (**C**) Immunoblotting analysis of MLH1 and ACTIN in WT and *Mlh1^−^^/^^−^* mESCs transfected with EV, MLH1-WT or MLH1-Δ746–756 (left). Also shown is immunoblotting analysis of FLAG-PMS2 and ACTIN in WT mESCs transfected with siCTRL EV or siPms2 with EV, PMS2-WT or PMS2-E702K (right). (**D**) Shown are RMD frequencies in WT mESCs transfected with either siCTRL EV or siPms2 with EV, PMS2-WT or PMS2-E702K. Frequencies are normalized to transfection efficiency and parallel siCTRL (= 1). *n* = 6. **P ≤ 0.005, ***P ≤ 0.0005, ****P < 0.0001, unpaired *t*-test with Holm−Sidak correction. Data are represented as mean values ± SD.

To begin with, we tested how siRNAs targeting PMS2 and PMS1 individually, and in combination, affect four distinct RMD events: the two divergent repeat assays (1% RMD-GFP and 3%RMD-GFP), each at two DSB/repeat distances (9.1 kb and 16 bp). We found that siRNAs targeting PMS1 caused a significant increase in RMDs at both 9.1 kb and 16 bp in the 1% divergent reporter, and at 16 bp in the 3% reporter, but not at 9.1 kb in the 3% divergent reporter (Figure 6A). We also found that siRNAs targeting PMS2 caused a significant increase in all four of these RMD events, where the fold-effects were either similar or greater than the effects of siRNAs targeting PMS1 (Figure 6A). Finally, combining siRNAs targeting PMS2 and PMS1 caused the greatest increase in all four of these RMD events that was significantly higher than depleting the two factors alone (Figure 6A). As controls, we also evaluated knockdown of PMS2 and PMS1 in the RMD assay with identical repeats and found largely no effect on RMDs (Supplemental Figure S7A). Furthermore, siRNAs targeting PMS2 and PMS1 had no effect on RMD frequencies in the *Mlh1^−^^/^^−^* mESCs (all of the identical and divergent repeat assays tested at 16 bp and 9.1 kb DSB/repeat distance, Supplemental Figure S7B). We confirmed knockdown of PMS2 and PMS1 transcript relative to siCTRL treated cells in both WT and *Mlh1^−^^/^^−^* mESCs via qRT-PCR (Supplemental Figure S7C, D). Altogether, these findings indicate that MLH1-PMS2 and MLH1-PMS1 have a role in MLH1-dependent suppression of divergent RMDs. Identifying a role for PMS1 in these events is somewhat unexpected, since it lacks nuclease activity, and its role in mismatch repair has been unclear (30,31). However, the notion that PMS1 might have a partial backup function with PMS2 is also supported by a study in *S. cerevisiae* that PMS1 (*S. cerevisiae MLH2*) suppresses mutation rates when combined with reduced expression of PMS2 (*S. cerevisiae PMS1*) (31).

Based on these effects of double knockdown of PMS1 and PMS2, we also tested combined knockdown of these two factors with another MLH1 binding partner: MLH3. A rationale for this experiment is that combined loss of these three MLH1 binding partners in *S. cerevisiae* showed a similar phenotype as loss of MLH1 using an assay for recombination between divergent sequences (40). In contrast, in our assay system, we found that combined knockdown of MLH3, PMS2 and PMS1 failed to cause an increase greater than that of the PMS2 and PMS1 double knockdown (Supplemental Figure S8A).

Given that MLH1-PMS2 has a role in suppressing divergent RMDs, we then considered that its nuclease domain might be important for this function. To test this hypothesis, we examined mutants of MLH1 and PMS2 that have been shown to disrupt endonuclease activity. We first tested an MLH1 mutant (Δ754–756) with the final three C-terminal amino acids deleted, which have been shown to reside in the metal binding domain that is critical for MLH1-PMS2 endonuclease activity, but are apparently dispensable for binding to PMS2 (27,60). We then compared RMD frequencies at 16 bp and 9.1 kb in the two divergent reporters in *Mlh1^−^^/^^−^* mESCs expressing either MLH1-WT or Δ754–756. We found that at both 16 bp and 9.1 kb, MLH1-Δ754–756 failed to reduce RMDs (Figure 6B). We confirmed both MLH1 WT and Δ754–756 expression via immunoblot (Figure 6C).

We also tested effects of expressing MLH1-WT and Δ754–756 in WT mESCs (Supplemental Figure S9A, S9B, S9C). For this analysis, we used 6 RMD events: (i) RMD-GFP with the 16 bp DSB/repeat distance, (ii) RMD-GFP with the 9.1 kb DSB/repeat distance, (iii) 1%RMD-GFP with the 16 bp DSB/repeat distance, (iv) 1% RMD-GFP with the 9.1 kb DSB/repeat distance, (v) 3%RMD-GFP with the 16 bp DSB/repeat distance and (vi) 3% RMD-GFP with the 9.1 kb DSB/repeat distance. We found that MLH1-WT expression caused a decrease for RMD-GFP at 16 bp, and an increase in 3% RMD-GFP at 9.1 kb, but no significant difference at the other four RMD events. Expression of MLH1-Δ754–756 caused an increase in all four RMDs with sequence divergence, but no significant effect on the identical repeat RMDs. Thus, while MLH1-WT expression did not cause an obvious/consistent pattern, expression of MLH1-Δ754–756 appears to have a consistent dominant negative effect, in that its expression caused an increase in RMDs with sequence divergence (Supplemental Figure S9A−C). These findings are consistent with the MLH1 C-terminal domain being important to suppress RMDs between divergent repeats.

We next tested a PMS2 mutant (E702K), which also disrupts the metal binding domain of MLH1-PMS2 (61). Specifically, we expressed siRNA resistant forms of PMS2 WT and E702K in cells treated with the siRNAs targeting PMS2. We examined the same four RMD events described above, and found that expression of PMS2 WT, but not E702K, inhibits RMDs between divergent repeats (Figure 6C, D). We also confirmed PMS2 WT and E702K expression via immunoblotting using a 3xFLAG immunotag (Figure 6C). In summary, these findings indicate that the endonuclease domain of MLH1-PMS2 is important for suppression of RMDs between divergent repeats.

### Resolution of sequence divergence in RMD products exhibits a polarity that is mediated by MLH1

**Figure 7.**
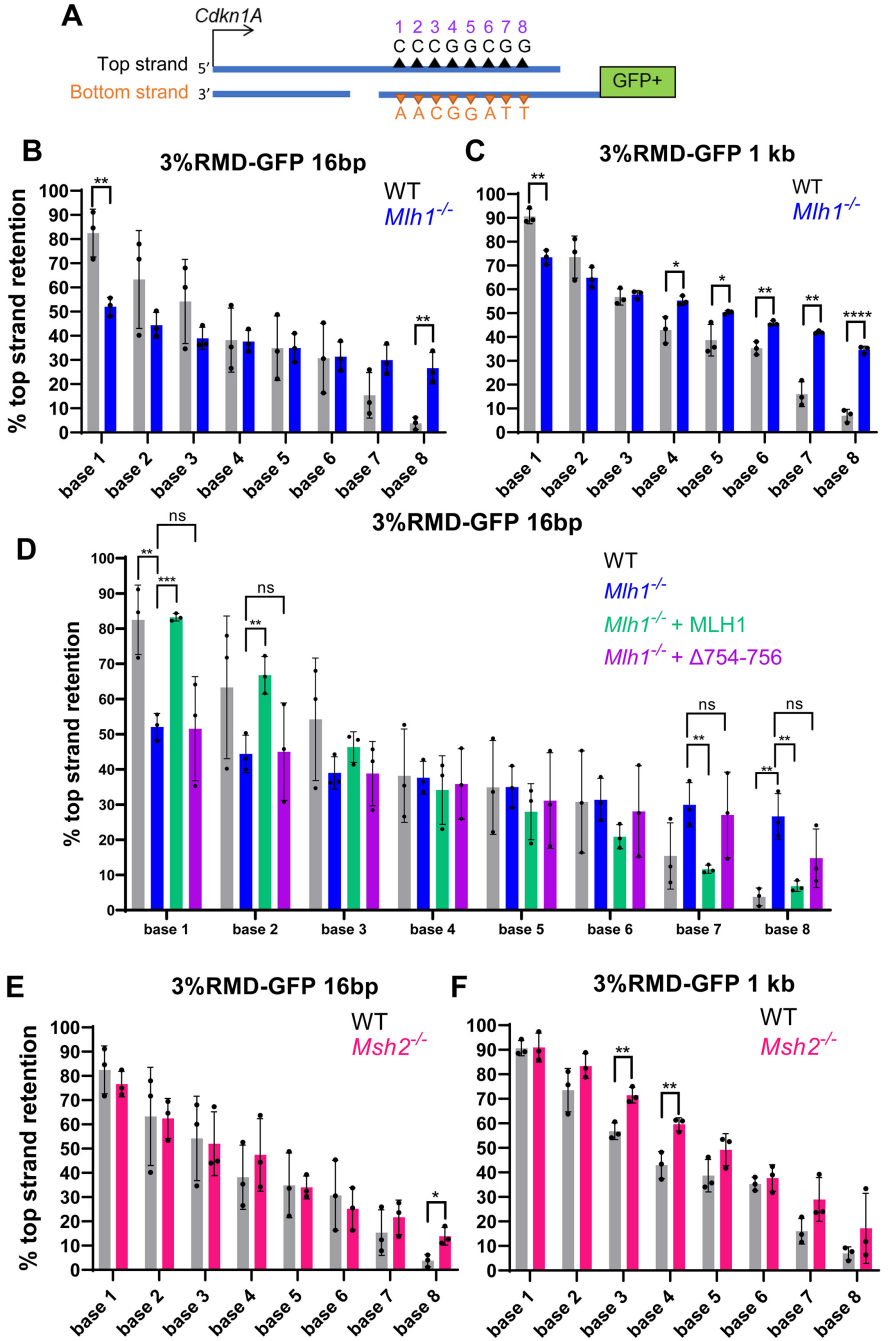
Resolution of sequence divergence in RMD products exhibits a polarity that is mediated by MLH1. (**A**) Shown is a diagram of an RMD annealing intermediate with the eight divergent bases of the top and bottom strand shown as triangles, which are numbered 1–8. The DSB ends are shown as DNA nicks without 3’ non-homologous tails only for simplicity. Resolution of sequence divergence in RMD products was determined by sorting GFP+ cells from the 3% RMD-GFP reporter, amplifying the repeat sequence, and performing deep sequencing analysis to determine the frequency of retention of the top strand base for each site. (**B**) Shown is the frequency of top strand base retention for WT and *Mlh1^−^^/^^−^* mESCs using the 16 bp DSB/repeat distance. *n* = 3. **P ≤ 0.005, unpaired *t*-test. (**C**) Shown is the analysis as in (B), except using the 1 kb DSB/repeat distance. *n* = 3. *P ≤ 0.05, **P ≤ 0.005, ****P < 0.0001, unpaired *t*-test. (**D**) Shown is the frequency of top strand base retention using the 16 bp DSB/repeat distance for WT, *Mlh1^−^^/^^−^* and *Mlh1^−^^/^^−^* transfected with expression vectors for MLH1 (WT) and MLH1-Δ754–756. Results for WT and *Mlh1^−^^/-^*are the same as in (B). *n* = 3. **P ≤ 0.005, ***P ≤ 0.0005, ns = not significant, *Mlh1^−^^/^^−^* versus *Mlh1^−^^/^^−^* + MLH1 (WT) and *Mlh1^−^^/^^−^* versus *Mlh1^−^^/^^−^* + MLH1-Δ754–756, unpaired *t*-test with Holm−Sidak correction. (**E**) Shown is the frequency of top strand base retention at 16 bp in WT and *Msh2^−^^/^^−^* mESCs. WT results are the same as in (A). *n* = 3. *P ≤ 0.05, unpaired *t*-test. (**F**) Shown is the analysis as in (E), but using the 1 kb DSB/repeat distance. WT results are the same as in (B). *n* = 3. **P ≤ 0.005, unpaired *t*-test. Data are represented as mean values ± SD.

We next considered that MLH1 might also influence the resolution of the RMD product. Specifically, based on the SSA model for RMDs, we considered whether MLH1-PMS2 might cleave the heteroduplex intermediate in a manner that affects the pattern of resolution of divergent bases in the RMD product. To address this hypothesis, we first tested whether resolution of divergent bases in the RMD product follows a specific pattern, or is random. We used the 3% RMD-GFP reporter to determine which base for each of the 8 mismatches was retained in the final RMD product (Figure 7A). We performed this reporter assay using two different 3’ DSB/repeat distances (16 bp and 1 kb) in WT mESCs, sorted the GFP + cells by flow cytometry, amplified the rearrangements, and performed deep sequencing analysis. Each of the 8 divergent bases were scored as having either the base from the 5’ repeat in the *Cdkn1A* locus (labeled as the top strand), or from the 3’ repeat fused to GFP (labeled as the bottom strand) (Figure 7A). We numbered the divergent bases 1–8 starting from the *Cdkn1A* side. We performed this analysis with three independent transfections/sorts for each condition to determine the mean/standard deviation for the frequency of retention of the base in the top strand.

We found that the retention of the top strand base showed a striking polarity in WT cells, for both the 16 bp and 1 kb 3’ DSB/repeat distances (Figure 7B, C). Specifically, on the *Cdkn1A* side there is preferential retention for the top strand base, whereas on the GFP side there is a preferential loss of the top strand base, and the bases in the middle show no strong bias for either base (Figure 7B, C). This polarity is supported by statistical comparisons (Supplemental Table S3). For example, the first base on the *Cdkn1A* side (base 1) shows significantly greater retention of the top strand, compared to bases 4 through 8. Conversely, the last base from the *Cdkn1A* side (base 8) shows significantly lower retention of the top strand, compared to bases 1 through 3. Based on the SSA model for RMDs, this pattern is consistent with preferential loss the bases proximal to DSB end, i.e. the bases closest to the 3’ non-homologous tail in the SSA annealing intermediate, or if the tail has been removed, then the bases closest to the DNA nick (Figure 7A, Supplementary Figure S1A).

We then examined the resolution of sequence divergence in the RMD products in the *Mlh1^−^^/-^*cell line, also with both the 16 bp and 1 kb DSB/repeat distances. We found that while the resolution of divergent bases still showed polarity, the degree of this polarity is markedly reduced, compared to WT (Figure 7B, C, Supplemental Table S3). For example, for both DSB/repeat distances in *Mlh1^−^^/^^−^* cells, base 1 exhibits higher strand retention versus bases 4–6, which was similar to WT (Supplemental Table S3). However, the frequency of top strand retention for base 1 was substantially lower for *Mlh1^−^^/^^−^* versus WT (Figure 7B, C). Conversely, the frequency of top strand retention for base 8 was substantially higher for *Mlh1^−^^/^^−^* versus WT at both DSB/repeat distances (Figure 7B, C, Supplemental Table S3). These data indicate that MLH1 promotes the polarity for resolution of sequence divergence in RMD products.

We next posited that the domain of MLH1 that forms part of the MLH1-PMS2 endonuclease (i.e. residues 754–756, as described above) is important for the polarity of resolution of the sequence divergence in the RMD products. To test this hypothesis, we performed the 3%RMD-GFP assay with the 16 bp DSB/repeat distance in *Mlh1^−^^/^^−^* mESCs with expression of MLH1-WT and MLH1-Δ754–756, and then examined the sequence of the RMD products, as described above. From these experiments, we found that expression of MLH1-WT, but not MLH1-Δ754–756, caused an increase in top strand retention for bases 1 and 2, and a converse reduction in top strand retention for bases 7 and 8 (Figure 7D). Thus, MLH1-WT expression, but not MLH1-Δ754–756, restored the polarity in divergent base resolution in the RMD products, indicating that the domain of MLH1 that forms part of the MLH1-PMS2 endonuclease is important for this polarity.

As MLH1 and MSH2 function in the same pathway for RMD suppression (Figure 5A), we also examined resolution of divergent bases in RMD products in *Msh2^−^^/^^−^* mESCs at both 16 bp and 1 kb. We found that *Msh2^−^^/^^−^* versus WT cells showed very few statistical differences for the frequency of top strand retention (Figure 7E, F). For the 16 bp DSB/repeat distance, only base 8 showed a statistical difference, with *Msh2^−^^/^^−^* mESCs showing an increase in top strand retention. Also, for the 1 kb DSB/repeat distance, bases 3 and 4 showed statistically higher retention of the top strand in *Msh2^−^^/^^−^* mESCs versus WT, which indicates that for these bases, the polarity was enhanced by loss of MSH2. In summary, WT cells have a polarity for resolution of divergent bases in RMD products, which is markedly reduced with loss of MLH1, or the domain of MLH1 that forms part of the MLH1-PMS2 endonuclease, whereas loss of MSH2 has a more modest effect.

### TOP3α suppresses RMDs in a manner that is distinct from MLH1

**Figure 8.**
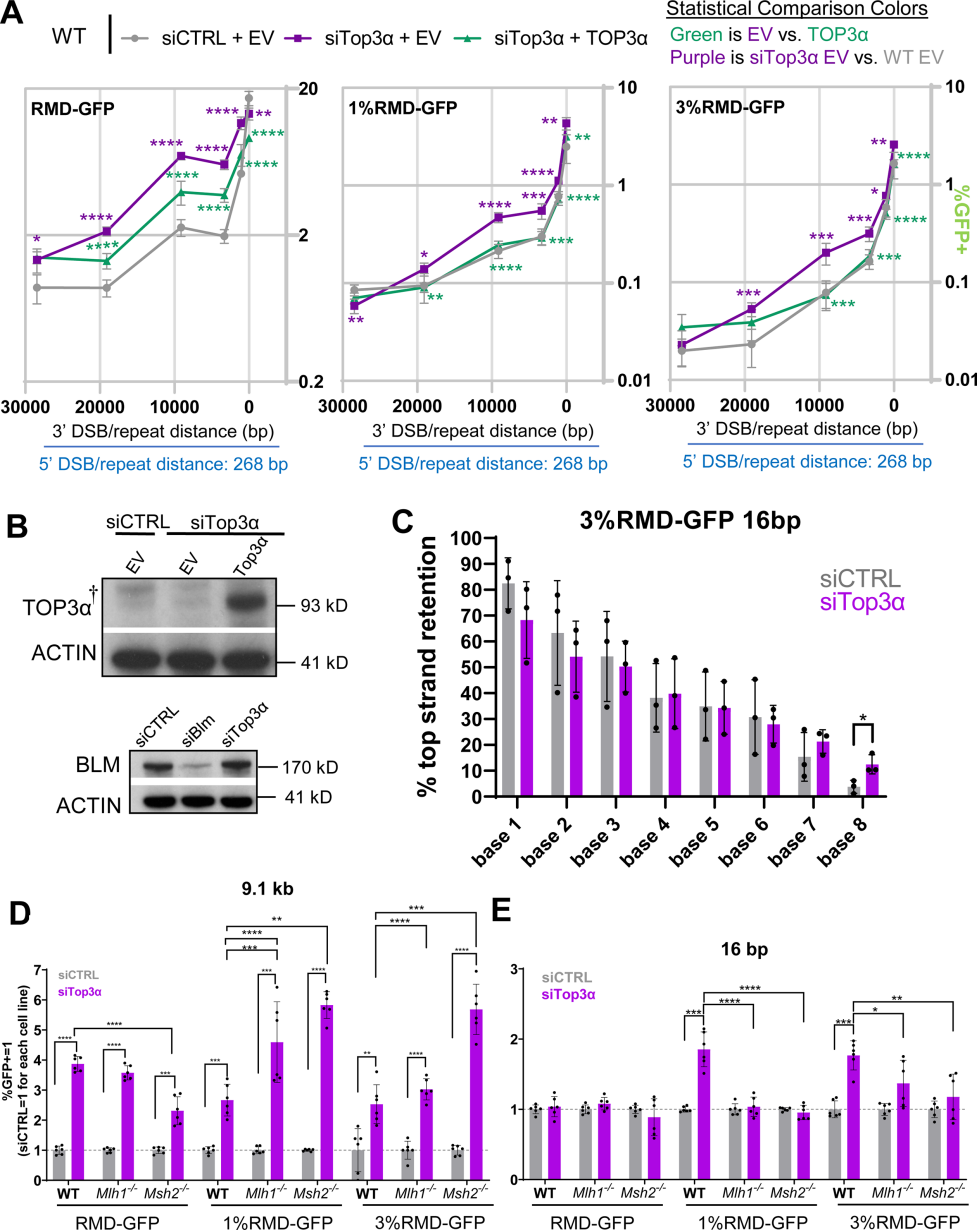
TOP3α suppresses RMDs in a manner that is distinct from MLH1. (**A**) Shown are the frequencies of the RMD events depicted in the diagram in Figure 2A (i.e. six different DSB/repeat distances with RMD-GFP, 1% RMD-GFP and 3% RMD-GFP) for WT mESCs transfected with siCTRL and EV, siTop3a and EV, and siTop3α and TOP3α expression vector. *n* = 6. *P ≤ 0.05, **P ≤ 0.005, ***P ≤ 0.0005, ****P < 0.0001, unpaired *t*-test with Holm−Sidak correction. (**B**) Immunoblotting analysis of TOP3α and ACTIN in WT mESCs transfected with either siCTRL EV, siTop3α EV or siTop3a with TOP3α expression vector. Endogenous mouse TOP3α was not detected, likely due to the immunogen being human TOP3α. Also shown is immunoblotting analysis of BLM and ACTIN in WT mESCs transfected with either siCTRL, siBlm or siTop3α. (**C**) Shown is the frequency of top strand base retention performed as in Figure 5B, at the 16 bp DSB/repeat distance for WT (siCTRL) and WT siTop3α. WT (siCTRL) values are the same as in Figure 5B. *n* = 3. *P ≤ 0.05, unpaired *t*-test. (**D**) Shown is the effect of siRNAs targeting TOP3α (siTop3α) on three RMD events (9.1 kb DSB/repeat distance, RMD-GFP, 1% GFP-GFP, 3% RMD-GFP) in WT, *Mlh1^−^^/^^−^* and *Msh2^−^^/^^−^*, mESCs. Frequencies are normalized to transfection efficiency and parallel siCTRL (= 1). *n* = 6. **P ≤ 0.005, ***P ≤ 0.0005, ****P < 0.0001, unpaired *t*-test for siCTRL versus siTop3α, and unpaired *t*-test using Holm−Sidak correction for effect of siTop3α in WT versus the other genetic backgrounds. (**E**) Shown is the analysis as in (D), but using the 16 bp DSB/repeat distance. *n* = 6. Statistics as in (D), except with **P ≤ 0.005. Data are represented as mean values ± SD. The † symbol notes that endogenous mouse Top3α is not readily detected by this antibody raised against the human protein.

Finally, we sought to contrast MLH1 with another factor that we identified in the siRNA survey as also suppressing RMDs: TOP3α. We performed each of the RMD assays with cells treated with siRNAs targeting TOP3α, which were also co-transfected with either a TOP3α expression vector with silent mutations to be siRNA-resistant, or EV (Figure 8A). Beginning with RMD-GFP, we found that knockdown of TOP3α lead to an increase in RMD events at all DSB/repeat distances except 16 bp (Figure 8A). Furthermore, expression of siRNA resistant TOP3α caused a decrease these events at all DSB/repeat distances except 28.4 kb. In both the divergent reporters (1% and 3%), disruption of TOP3α caused a significant increase in RMDs at all DSB/repeat distances except 28.4 kb, and these effects were reversed with the TOP3α expression vector, except for the 19 kb DSB with 3%RMD-GFP (Figure 8A).

We next confirmed expression of TOP3α using immunoblot analysis (Figure 8B), examined a catalytically dead mutant of TOP3α (Y362F) (62), and tested effects of TOP3α on resolution of sequence divergence in the RMD product. In addition, we tested whether TOP3α knockdown affected the level of BLM protein, and found no obvious effect (Figure 8B). We found that while TOP3α WT expression can suppress a set of RMDs, the Y362F mutant had no effect (Supplemental Fig S10A). However, with immunoblot analysis, we found that the TOP3α-Y362F mutant had a much lower molecular weight, which is consistent with other reports of TOP3α mutants that are prone to degradation (Supplemental Fig S10B) (63). We tested resolution of sequence divergence in final RMD products in cells treated with TOP3α siRNA using the 16 bp DSB/repeat distance, finding that the polarity in resolution of divergent bases in RMD products was not obviously affected, with only a slight increase in top strand retention at base 8 (Figure 8C).

Using the RMD frequency data, we then compared the fold-effects of TOP3α knockdown among the various degrees of repeat divergence (identical, 1%, and 3%), and for each DSB/repeat distance. We found that RMDs with identical repeats (RMD-GFP) were effected to at least the same degree as the divergent repeat RMDs by TOP3α knockdown, except for the 16 bp 3’ DSB/repeat distance (Supplemental Figure S11A). Namely, with the 16 bp DSB, knockdown of TOP3α lead to a significant increase in RMD events for the divergent repeats, but not for the identical repeats. With regards to effect of DSB/repeat distance, we found that TOP3α knockdown caused different fold-effects dependent on DSB/repeat distance (Supplemental Figure S11B). The most striking difference is with the RMD-GFP assay, for which TOP3α knockdown caused a marked increase in RMDs at both 9.1 and 3.3 kb, which was statistically higher than 19.1 and 1 kb, which themselves were statistically higher than 28.4 kb and 16 bp (Supplemental Figure S11B). The effects of DSB/repeat distance with the divergent repeat RMDs was similar, but more modest (Supplemental Figure S11B).

The above findings indicate that the types of RMDs suppressed by TOP3α are distinct from those of MLH1, which led us to hypothesize that loss of these factors may function independently for RMD suppression. Thus, we examined whether knockdown of TOP3α caused further increases in RMDs in *Mlh1^−^^/^^−^* mESCs for the 16 bp and 9.1 kb DSB/repeat distances. We also tested *Msh2^−^^/^^−^* for comparison. Knockdown of TOP3α caused a marked increase in RMDs with the 9.1 kb DSB in WT, *Mlh1^−^^/^^−^* and *Msh2^−^^/^^−^* mESCs, for both identical and divergent repeats (Figure 8D). Interestingly, with the 16 bp DSB and with divergent repeats, knockdown of TOP3α only caused an increase in RMDs in WT mESCs, but failed to do so in *Mlh1^−^^/^^−^*, and *Msh2^−^^/^^−^* (Figure 8E). We confirmed knockdown of the TOP3α RNA in each of the cell lines (Supplemental Figure S11C). These results indicate that TOP3α suppresses RMDs in a manner that is independent to MLH1 and MSH2 when the DSB/repeat distance is long, but is in the same pathway when the DSB/repeat distance is short.

## DISCUSSION

To characterize factors that regulate RMDs, we began with a survey of several DNA damage response factors in mouse cells, and identified 19 different factors that affect the frequency of RMDs, including several mismatch repair factors and components of the BTR complex that suppress RMDs. We then focused largely on MLH1, which we found suppresses RMDs, but only when the repeats contained sequence divergence. Indeed, the fold-suppression of RMDs via MLH1 increases along with sequence divergence. We also found that MLH1 acts in the same pathway to the MSH2-MSH6 complex, and two MLH1 binding partners (PMS2 and PMS1) for suppression of such RMDs. Finally, we found that the endonuclease domain of the MLH1-PMS2 complex is important to suppress such RMDs. Notably, our findings are consistent with a recent study that the domain of MLH1 that forms part of the endonuclease domain of MLH1-PMS2 suppresses prime editing in human cells (i.e. recombination events induced by a DNA nick that use a localized reverse transcribed DNA template for gene editing) (64).

**Figure 9.**
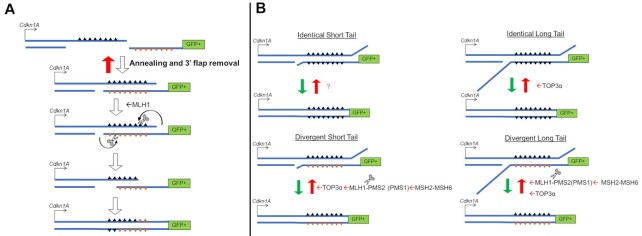
Model. (**A**) Model for the polarity of resolution of sequence divergence in RMDs, which is promoted by the domain of MLH1 that contributes to the MLH1-PMS2 endonuclease. (**B**) Model for RMD suppression for short and long DSB/repeat distances (and hence short versus long 3’ non-homologous tails, respectively), with and without sequence divergence between repeats.

Apart from suppression of RMDs, we also found that MLH1 is important for the pattern of resolution of sequence divergence in RMDs (Figure 9A). Specifically, in WT cells we found preferential retention of the top strand base on the *Cdkn1A* side, whereas on the GFP side there is a preferential retention of the bottom strand base (Figure 7B, C). Accordingly, WT cells show a polarity for resolution of sequence divergence in the RMD product. Evidence of polarity in homologous recombination between divergent sequences has been found in other circumstances. For one, analysis of gene conversion events from meiotic recombination in *S. cerevisiae* found evidence of polarity gradients, which refers to preferential gene conversion near the ends of genes (13,65). Such polarity gradients are dependent on several components of mismatch repair, including PMS1 (PMS2 in mammalian cells) and MSH2 (13). As another example, Alu-Alu RMDs show polarity in recombination junctions. Namely, the recombination junction for Alu-Alu RMDs are biased towards the 5’ end of Alu elements (2,7,66). It is unclear whether the polarity phenomenon described here for RMDs is related to the polarity observed with Alu-Alu RMDs or during meiotic recombination in *S. cerevisiae*.

We found that the polarity for resolution of sequence divergence in the RMD product is largely dependent on MLH1 and the endonuclease domain of MLH1-PMS2 (i.e. the polarity failed to be restored with the MLH1-Δ754–756 mutant). Considering the SSA model for these events, sequence divergence causes mismatched bases in the annealed repeats (Supplemental Figure S1A, Figure 9A). Accordingly, we propose a model whereby MLH1 creates an incision upstream of mismatched bases on the strand that is proximal to a DSB end, which initiates degradation and/or replication displacement of the incised strand, and hence loss of the mismatched bases on the strand proximal to the DSB end (Figure 9A). The bias towards creating an incision proximal to the DSB end could be mediated by the 3’ non-homologous tail in the annealing intermediate, or if the tail has been removed, then the resulting the DNA nick (Figures 7A and 9A). Notably, induction of an incision upstream from a DNA nick is similar to models of mismatch repair at the replication fork. Specifically, components of the replisome (i.e. PCNA and RFC) and MSH2-MSH6 appear to direct MLH1-PMS2 to cleave nicked heteroduplex DNA with a strand bias to the nicked DNA strand (67–69). These studies with purified proteins support a model of replisome-directed incision of heteroduplex DNA via MLH1-PMS2 that is biased to the nascent strand due to the presence of a DNA nick at the 3’ end of the nascent strand. Consistent with this model, overexpression of DNA ligase in *S. cerevisiae* causes an increase in mutation rates, and hence reduced mismatch repair, which appears to be caused by premature loss of the DNA nick on the nascent strand (70). While this polarity for mismatch repair with purified proteins is consistent with the polarity we observe with RMDs in mouse cells, the mechanisms may not be precisely the same.

Indeed, there are apparent distinctions between mismatch resolution with purified proteins versus the RMDs measured in our study, due the findings with MSH2. Namely, while MSH2 is important to direct MLH1-PMS2 to cleave nicked heteroduplex DNA (67,68), MSH2 had a more modest role versus MLH1 on the resolution of sequence divergence in the RMD product. We speculate that MLH1 may be directly recruited to DNA nicks or 3’ non-homologous tails in the SSA intermediate to cleave upstream from mismatched bases that are proximal to the DSB end (Figure 7A). These findings are consistent with the intrinsic nuclease activity of MLH1 in complex with PMS2, although certainly this activity is markedly activated with inclusion of other factors (e.g. MSH2, MSH6, PCNA, and RFC) (34,60). Another implication of these findings is that suppression of RMDs between divergent repeats versus mismatch resolution appear to have distinct mechanisms. Namely, as mentioned above, the effects of MLH1 in suppressing RMDs between divergent repeats are in the same pathway with MSH2 and MSH6. Altogether, we suggest that MLH1 has multiple roles in regulation of RMDs between divergent repeats: both suppression of these events in a manner that is in the same pathway as MSH2-MSH6, and also an independent role in resolution of sequence divergence in RMD products to promote the preferential loss of the divergent base near the chromosomal break end.

Future studies could focus on defining how other aspects of mismatch repair affect the polarity of resolution of sequence divergence, including the mechanisms of excision subsequent to MLH1-PMS2 cleavage, which could involve EXO1 or EXO1-independent pathways, such as involving RAD27/FEN1, iterative nicking via MLH1-PMS2, and/or displacement synthesis (36–39). Along these lines, it will be interesting to examine DNA polymerase delta proofreading activity, which has been found to influence resolution of sequence divergence in recombination events in *S. cerevisiae* (71–73). Additionally, future studies could focus on consequences of MLH1-PMS2 cleavage of divergent recombination substrates on genome stability. Namely, one possible consequence of iterative nicking via MLH1-PMS2 could be destruction of the annealing intermediate, which could possibly lead to persistent breaks and chromosome loss, and/or reliance on end joining pathways to restore the chromosome. Consistent with this latter possibility, large deletions that were likely caused by such end joining were observed with DSB reporter assays using divergent Alu sequences (66).

Regarding suppression of divergent RMDs, we also found that PMS1 appears to play a role in this process. The role of PMS1 in suppressing such RMDs, and indeed mismatch repair, remains poorly understood, because PMS1 does not appear to contain a functional nuclease domain (59). One possibility is that PMS1 may play a structural role in facilitating MLH1-PMS2 endonuclease activity. Consistent with this notion, we found that combining siRNAs targeting PMS2 and PMS1 caused the greatest increase in divergent RMDs, versus depleting the two factors alone. Similarly, in *S. cerevisiae*, loss of MLH2 (mammalian PMS1) was shown to cause an increase in mutation frequencies in combination with knockdown of PMS1 (mammalian PMS2) (31).

Finally, we also found marked distinctions between MLH1 versus TOP3α in suppression of RMDs. For one, nearly all of the RMD events we examined are suppressed by TOP3α, largely irrespective of sequence divergence or DSB/repeat distance. Interesting exceptions include the short DSB/repeat distance (16 bp) for RMDs with identical repeats, as well as RMDs with the longest DSB/repeat distance (28.4 kb). Accordingly, TOP3α appears to have a relatively promiscuous anti-RMD activity, which is distinct from the influence of MLH1, which is dependent on sequence divergence. Furthermore, the effects of TOP3α were independent of MLH1 (and MSH2) for the 9.1 kb DSB/repeat distance. These findings are consistent with reports that TOP3α is important to suppress recombination between divergent sequences in *S. cerevisiae*, and that combined loss of another BTR component (*SGS1*/BLM) and *MSH2* causes an increase in such recombination that is greater than the single disruptions both in *S. cerevisiae* and the mESC assay system described here (55,74).

However, interestingly TOP3α appears to function in the same pathway with MLH1 and MSH2 for suppressing divergent RMDs with a short DSB/repeat distance of 16 bp. A likely consequence of a short DSB/repeat distance is the lack of a long non-homologous 3’ tail in the annealing intermediate during SSA. Thus, we suggest that TOP3α functions independently of mismatch repair to suppress RMDs when there is a long 3’ non-homologous tail (e.g. 9.1 kb DSB/repeat distance), but is mediated by mismatch repair with a short tail (16 bp DSB/repeat distance, and only with sequence divergence) (Figure 9B). In contrast, we did not observe an obvious effect of DSB/repeat distance on the relative role of MLH1 or MSH2 on suppression of RMDs. However, it will be important to develop assays that have no DSB/repeat distance (i.e. no 3’ non-homologous tail), since studies in *S. cerevisiae* indicate that even a short 3’ non-homologous tail is important to signal suppression of divergent sequence recombination via MSH2 (73).

As mentioned above, the role of TOP3α in suppressing RMDs is likely linked to its role in the BTR complex, since knockdown of BLM, RMI1, and RMI2 each had similar effects (e.g. each suppress RMDs with identical repeats with the 9.1 kb DSB/repeat distance, but not 16 bp). The BTR complex has been shown to resolve diverse DNA structures (75), which likely accounts for its robust anti-RMD activity. The catalytic activity of TOP3α may also be important for suppressing RMDs, but our experiments with the Y362F mutant were inconclusive because the mutant protein migrates at a lower molecular weight, which is consistent with a report that mutants of TOP3α are prone to degradation (63). In summary, whereas MLH1 specifically suppresses RMDs between divergent repeats and also mediates the polarity of resolution of sequence divergence in RMD products, TOP3α suppresses a diverse set of RMDs, which is in the same pathway as MLH1 and MSH2 when the repeats have sequence divergence, and the DSB/repeat distance is short.

## DATA AVAILABILITY

The data underlying this article are available in the article and in its online supplementary material, which includes deep sequencing source data used for the analysis of resolution of sequence divergence in the RMD product.

## Supplementary Material

gkac1240_Supplemental_Files
