# Supplementary material for: Resolution of sequence divergence for repeat-mediated deletions shows a polarity that is mediated by MLH1
Source: Nucleic Acids Res. 2023 Jan 9;51(2):650–67. doi: 10.1093/nar/gkac1240 (PMC9881173; doi:10.1093/nar/gkac1240)
Supplement: gkac1240_Supplemental_Files [file gkac1240_supplemental_files.zip › TrostSupplementalMaterials117.pdf]

SUPPLEMENTAL INFORMATION

Resolution of sequence divergence for repeat-mediated deletions show a polarity that is mediated by MLH1

Hannah Trost, Arianna Merkell,
Felicia Wednesday Lopezcolorado, Jeremy M. Stark

Supplementary Table S1: siRNA list

Pool Catalog Number	Duplex Catalog Number	Gene Symbol	GENE ID	Gene Accession	Sequence
M-047552-01	D-047552-01	Top3a	21975	NM_009410	GAACAUCGGCUUUGAGAUU
M-047552-01	D-047552-02	Top3a	21975	NM_009410	CCACAAAGAUGGGACCGUA
M-047552-01	D-047552-03	Top3a	21975	NM_009410	GACAUUUGCUGGCUCAUGA
M-047552-01	D-047552-04	Top3a	21975	NM_009410	GAGAUGAUCGGCGACUGUA
M-058750-01	D-058750-01	Ep400	75560	NM_173066	AAACCGACCUUUCUAUAUU
M-058750-01	D-058750-02	Ep400	75560	NM_173066	CAUGAGGAGUUGUAACAUA
M-058750-01	D-058750-03	Ep400	75560	NM_173066	GGACGGUUCUCAGGAUAAA
M-058750-01	D-058750-04	Ep400	75560	NM_173066	CUAAGAAGCUUGUCAGAAC
M-064499-01	D-064499-01	Chaf1b	110749	NM_028083	CACCAAAGCUGUCAAUGUU
M-064499-01	D-064499-02	Chaf1b	110749	NM_028083	GCAAGGAGCCGGAGCAGAU
M-064499-01	D-064499-03	Chaf1b	110749	NM_028083	UGACAAACACCACUUAUGU
M-064499-01	D-064499-04	Chaf1b	110749	NM_028083	GGGCAACCGAUGGGAAUUU
M-063756-01	D-063756-21	Zswim7	69747	XM_001473892	GCUAGGGAGUUCUGGCAAA
M-063756-01	D-063756-22	Zswim7	69747	XM_001473892	CCUCUAUGGUCUUAAUAUU
M-063756-01	D-063756-23	Zswim7	69747	XM_001473892	GUAAGCAUCUUCUGGCAAU
M-063756-01	D-063756-24	Zswim7	69747	XM_001473892	CCUUCUCAGUGUUACGGAA
M-058186-01	D-058186-01	Fancm	104806	NM_178912	GAUGAGGACAUACGAUAUA
M-058186-01	D-058186-02	Fancm	104806	NM_178912	GGAAACAGGGCUUGCAUUC
M-058186-01	D-058186-03	Fancm	104806	NM_178912	AAGAAAGAAUGCUGGUAUU
M-058186-01	D-058186-04	Fancm	104806	NM_178912	GGUUACAAGUAGAGAUUUG
M-045170-01	D-045170-01	Ercc1	13870	NM_007948	GUGAGGUGAUUCCCGAUUA
M-045170-01	D-045170-02	Ercc1	13870	NM_007948	GAACUUCGCCCUUCGUGUG
M-045170-01	D-045170-03	Ercc1	13870	NM_007948	GGCGGUACCUGGAGACCUA
M-045170-01	D-045170-04	Ercc1	13870	NM_007948	UGUUGAAGUUUGUGCGCAA
M-049323-00	D-049323-01	Mlh1	17350	NM_026810	GCGACAAGGUCUACGCUUA
M-049323-00	D-049323-02	Mlh1	17350	NM_026810	GAGAUGCUCCGUAACCAUU
M-049323-00	D-049323-03	Mlh1	17350	NM_026810	CGGAGAUGCUUGCAGACUA
M-049323-00	D-049323-04	Mlh1	17350	NM_026810	CAAGUGAAGAGUACGGAAA
M-044082-00	D-044082-01	Msh6	17688	NM_010830	CUAGAAGGAUUCAAAGUAA
M-044082-00	D-044082-02	Msh6	17688	NM_010830	GCAUGAGGCUUAUUUAUCU
M-044082-00	D-044082-03	Msh6	17688	NM_010830	CGGAGGAGGUUAUUCAGAA
M-044082-00	D-044082-04	Msh6	17688	NM_010830	GCUAUAAUGUACGAAGAAA
M-048522-01	D-048522-01	Pms2	18861	NM_008886	GGACUCGACUGGUGUUUGA
M-048522-01	D-048522-02	Pms2	18861	NM_008886	GAAGUGGCCAGUAGCUUUA
M-048522-01	D-048522-03	Pms2	18861	NM_008886	CGUGUAAGCUGCACUAAUC
M-048522-01	D-048522-17	Pms2	18861	NM_008886	CCACAAAUGCCAAGCGCUU
M-049174-01	D-049174-01	Xrcc3	74335	NM_028875	GAGAAGAUCAGGUUCAGCA
M-049174-01	D-049174-02	Xrcc3	74335	NM_028875	CCGAAUUACUGCUGCGGUU
M-049174-01	D-049174-03	Xrcc3	74335	NM_028875	AAGACAAUAUGGAGGCCUA
M-049174-01	D-049174-04	Xrcc3	74335	NM_028875	UGUUGGAGUGUGUGAGUA A
M-054715-00	D-054715-01	Pms1	227099	NM_153556	UAACAGAGAGUCUUCUUAA
M-054715-00	D-054715-02	Pms1	227099	NM_153556	GAAUAUCGAACCUGUGAAA
M-054715-00	D-054715-03	Pms1	227099	NM_153556	GUACAUAAUAAGGCAGUUA
M-054715-00	D-054715-04	Pms1	227099	NM_153556	AAAGUUAAUUCGACGCUAU
M-041643-00	D-041643-01	Smarcal1	54380	NM_018817	GGAGUUAGCUUUGCCAUAU
M-041643-00	D-041643-02	Smarcal1	54380	NM_018817	GGAAGAACGUUCAACACAU
M-041643-00	D-041643-03	Smarcal1	54380	NM_018817	GGGGAUCGCUUCCGGGUAA
M-041643-00	D-041643-04	Smarcal1	54380	NM_018817	CAUGUGUCGUCGAGUAUAU

M-056921-01	D-056921-01	Fancd2	211651	NM_001033244	GCUAAAGACCGUUCGCUUA
M-056921-01	D-056921-02	Fancd2	211651	NM_001033244	GCAUGUGUGUUUAAAGUAU
M-056921-01	D-056921-03	Fancd2	211651	NM_001033244	UCUAUGGACUGGAAGAGUA
M-056921-01	D-056921-04	Fancd2	211651	NM_001033244	ACACAAGACUCACCAAGCA
M-045003-00	D-045003-01	Ercc4	50505	NM_015769	GGAGCGUGCUUCCGCCAAA
M-045003-00	D-045003-02	Ercc4	50505	NM_015769	GCCUGAAGUUGUAGAGAUU
M-045003-00	D-045003-03	Ercc4	50505	NM_015769	UCACAACCCGUCACUUGAA
M-045003-00	D-045003-04	Ercc4	50505	NM_015769	GCCGAAUACUCGUGGUUGA
M-058407-01	D-058407-01	Fanca	14087	NM_016925	UGACAGACCCGACCCAAUA
M-058407-01	D-058407-02	Fanca	14087	NM_016925	GGAGUCAGUUGGCCGUUUG
M-058407-01	D-058407-03	Fanca	14087	NM_016925	GACUAAGUGUCCCGUGAUU
M-058407-01	D-058407-04	Fanca	14087	NM_016925	GUCGGUGGAUGAGAUGUUA
M-059440-01	D-059440-01	Topbp1	235559	NM_176979	GGAUGAAUCUAUAUACAAG
M-059440-01	D-059440-02	Topbp1	235559	NM_176979	CCACGAUGGUUGAAGCUAA
M-059440-01	D-059440-03	Topbp1	235559	NM_176979	GCAGACAAUUACCGUGUAU
M-059440-01	D-059440-04	Topbp1	235559	NM_176979	GGAGUUUGCCGGCAGUUAA
M-057081-01	D-057081-01	Slx4	52864	NM_177472	CAUCUGAACUGUCACAAAU
M-057081-01	D-057081-02	Slx4	52864	NM_177472	GAGCUGCGUUUGAGUAUUC
M-057081-01	D-057081-03	Slx4	52864	NM_177472	GCGAGAUGCCCACUUCUUA
M-057081-01	D-057081-04	Slx4	52864	NM_177472	GCCCUAAGGUUUGGUGUGA
M-054618-01	D-054618-01	Pif1	208084	NM_172453	GAGCCUAGGUCGAAACGAA
M-054618-01	D-054618-02	Pif1	208084	NM_172453	CCAAUGGAAUCCCAGACUA
M-054618-01	D-054618-03	Pif1	208084	NM_172453	GUACGGUUCCUGUGUGGUA
M-054618-01	D-054618-04	Pif1	208084	NM_172453	GCCGUGUCCUUCAGUUAAA
M-055235-01	D-055235-01	Mus81	71711	NM_027877	GGACAUUGGCGAAACCAGA
M-055235-01	D-055235-02	Mus81	71711	NM_027877	GCAUUGCGCUCCCUCCAAC
M-055235-01	D-055235-03	Mus81	71711	NM_027877	GUGAAGCGAACCAUGGAUA
M-055235-01	D-055235-04	Mus81	71711	NM_027877	GUACGCAAGCUACACGUUG
M-053624-01	D-053624-01	Eme1	268465	NM_177752	AAUCAGGAAUGGCAAAUAA
M-053624-01	D-053624-02	Eme1	268465	NM_177752	GAGCAAGAACGUCAGAAUU
M-053624-01	D-053624-03	Eme1	268465	NM_177752	GCACGGGACUCAUGGUGUC
M-053624-01	D-053624-04	Eme1	268465	NM_177752	GGAUCUACCUUCAAAUGAC
M-046305-01	D-046305-01	Pold3	67967	NM_133692	AGAACAAGAUCGUGACUUA
M-046305-01	D-046305-02	Pold3	67967	NM_133692	UGGCUAAGCUAUACACUAG
M-046305-01	D-046305-03	Pold3	67967	NM_133692	CGAGUAGACUUGUCGGAUG
M-046305-01	D-046305-04	Pold3	67967	NM_133692	ACGAAAGCGUGUACUGAAA
M-059205-01	D-059205-01	Phf8	320595	NM_177201	GAUGAAGAGUGUAUCCAAA
M-059205-01	D-059205-02	Phf8	320595	NM_177201	CGGGAUAGCUAUACAGAUU
M-059205-01	D-059205-03	Phf8	320595	NM_177201	GGAAUUCUUGAUCUACUUA
M-059205-01	D-059205-04	Phf8	320595	NM_177201	GGAAAAGGCGAACCUGUUA
M-047716-00	D-047716-01	Msh5	17687	NM_013600	GCAUAGAUCAAGACACUUA
M-047716-00	D-047716-02	Msh5	17687	NM_013600	GAAAUCAUACUUCUGCCAA
M-047716-00	D-047716-03	Msh5	17687	NM_013600	GGACUACGGCUAUUCGAGA
M-047716-00	D-047716-04	Msh5	17687	NM_013600	CCGCAUUACUCUCCCUGUA
M-062301-01	D-062301-01	Mcm3ap	54387	NM_019434	CAAGACAGCUCACCAGAUA
M-062301-01	D-062301-02	Mcm3ap	54387	NM_019434	GCAAGAAGCUCGCCGUGAU
M-062301-01	D-062301-03	Mcm3ap	54387	NM_019434	GCGACAAACUUCCUCAAUU
M-062301-01	D-062301-04	Mcm3ap	54387	NM_019434	GUAUAAAGGUGGCUCAUGG
M-049912-00	D-049912-01	Rpa1	68275	NM_026653	UGAACAAGGUGUAUUACUU
M-049912-00	D-049912-02	Rpa1	68275	NM_026653	GGCUAAAGACGCACUAGUA
M-049912-00	D-049912-03	Rpa1	68275	NM_026653	GCUGAAGACGUUGGAUUAA
M-049912-00	D-049912-04	Rpa1	68275	NM_026653	CGCAUGAUCUUAUCGGCAA
M-055247-01	D-055247-01	Atrip	235610	NM_172774	ACGCAGAAAUGAAUGAGUU
M-055247-01	D-055247-02	Atrip	235610	NM_172774	GAGCAACGGACGAACAAAU

M-055247-01	D-055247-03	Atrip	235610	NM_172774	UCAUAAGGUCCGCCGAUUA
M-055247-01	D-055247-04	Atrip	235610	NM_172774	GGUGAGCACGCGGAAUUUA
M-056219-01	D-056219-01	Smarcad1	13990	NM_007958	CCACACAUGUUUAGCAGUA
M-056219-01	D-056219-02	Smarcad1	13990	NM_007958	GUUUAAAUGUGCUCUGUUA
M-056219-01	D-056219-03	Smarcad1	13990	NM_007958	GCAAUGUCAUGAUGCAAUU
M-056219-01	D-056219-04	Smarcad1	13990	NM_007958	GCACGUAACCGUUUAUUGC
M-040694-01	D-040694-01	Arid1a	93760	NM_001080819	GAAAUGACAUGACCUACAA
M-040694-01	D-040694-02	Arid1a	93760	NM_001080819	CCACCAGGCUACCCAAAUA
M-040694-01	D-040694-03	Arid1a	93760	NM_001080819	GAAUACCUCUGACAUGAUG
M-040694-01	D-040694-17	Arid1a	93760	NM_001080819	UUUAUAGUAUGGCGAGUUA
M-062392-02	D-062392-17	Setd2	235626	NM_001081340	CGAAAGAGAUCGAAGGCGA
M-062392-02	D-062392-18	Setd2	235626	NM_001081340	GGGAAAUCAUCAAGAUCGA
M-062392-02	D-062392-19	Setd2	235626	NM_001081340	CUCCAAUUGUGCAGAGUUA
M-062392-02	D-062392-20	Setd2	235626	NM_001081340	GAGUGGAGUAUGAGCGGAA
M-042994-01	D-042994-01	H2afz	51788	NM_016750	ACAAAUCGCUGAUCGGGAA
M-042994-01	D-042994-02	H2afz	51788	NM_016750	CAUCGACACCUGAAAUCUA
M-042994-01	D-042994-03	H2afz	51788	NM_016750	GUCAAAAGACUUAAAGGUA
M-042994-01	D-042994-04	H2afz	51788	NM_016750	UGCUAUACGUGGAGAUGAA
M-044077-01	D-044077-01	Msh3	17686	NM_010829	GAACUGCAGUACUUAGAUA
M-044077-01	D-044077-02	Msh3	17686	NM_010829	UAUACGAAAUCCACACUUA
M-044077-01	D-044077-03	Msh3	17686	NM_010829	AACUGAAACUGCCGCAUUA
M-044077-01	D-044077-04	Msh3	17686	NM_010829	GAUCCAAGAGCGUCUAUAC
M-046249-01	D-046249-01	Msh4	55993	NM_031870	GCGCAUGCCUGUACUCUUU
M-046249-01	D-046249-02	Msh4	55993	NM_031870	GCACCCAAAUCGCUAAAGA
M-046249-01	D-046249-03	Msh4	55993	NM_031870	GAACGUUAAUUUCACUACA
M-046249-01	D-046249-04	Msh4	55993	NM_031870	AGAGAUAGUGGACGACAUA
M-066289-01	D-066289-09	Mlh3	217716	NM_175337	GCUGAGAGCUUAGCCGUUA
M-066289-01	D-066289-10	Mlh3	217716	NM_175337	UGACAGGACUUAGCACAUU
M-066289-01	D-066289-11	Mlh3	217716	NM_175337	GAGGAACGAUGUAUCUGGA
M-066289-01	D-066289-12	Mlh3	217716	NM_175337	CAAAGAAGAUCGCUUAGAA
M-062824-01	D-062824-01	Mcm9	71567	NM_027830	GGUACUGGCUGGUGGAAUU
M-062824-01	D-062824-02	Mcm9	71567	NM_027830	CGAGCGGGAUUACAUGUGU
M-062824-01	D-062824-03	Mcm9	71567	NM_027830	CCACGGUCUAUGAAAGUUA
M-062824-01	D-062824-04	Mcm9	71567	NM_027830	AGAUGUACGCUGUGAAGUU
M-060852-01	D-060852-01	Asf1a	66403	NM_025541	CUACCGAGGUCAAGAAUUU
M-060852-01	D-060852-02	Asf1a	66403	NM_025541	AGAGUUGGCUACUAUGUAA
M-060852-01	D-060852-03	Asf1a	66403	NM_025541	UCGAGGACCUGUCUGAAGA
M-060852-01	D-060852-04	Asf1a	66403	NM_025541	AAUCUACAGUCCCUUCUUU
M-046122-01	D-046122-01	Asf1b	66929	NM_024184	GGGCUACUAUGUCAACAAU
M-046122-01	D-046122-02	Asf1b	66929	NM_024184	CAGUUGCACUCCUGUUAAA
M-046122-01	D-046122-03	Asf1b	66929	NM_024184	GUGAGGAGUUUGAUCAGAU
M-046122-01	D-046122-04	Asf1b	66929	NM_024184	UCUCUCAGCUACAGCGGAA
M-055156-00	D-055156-01	Brip1	237911	NM_178309	GGUUAUGAGUCGUCUUGUA
M-055156-00	D-055156-02	Brip1	237911	NM_178309	GUACGGACCAUUGUUCUAA
M-055156-00	D-055156-03	Brip1	237911	NM_178309	GACGGCAUUUCAAUAGUAA
M-055156-00	D-055156-04	Brip1	237911	NM_178309	CAACUCAAGUCGUGCUCAA
M-044778-01	D-044778-01	Recql	19691	NM_023042	GCAAAUCCAUGGAGAAUUA
M-044778-01	D-044778-02	Recql	19691	NM_023042	GUAAAGACGUUUCGUUUGA
M-044778-01	D-044778-03	Recql	19691	NM_023042	CAAGUGUCGCCGUGUGUUA
M-044778-01	D-044778-04	Recql	19691	NM_023042	UUAUAAGGCUCUUGGCAUC
M-058494-01	D-058494-01	Wrn	22427	NM_001122822	GAAUGACUCCUCCUAUAUA
M-058494-01	D-058494-02	Wrn	22427	NM_001122822	CGAAUCAUCUUGUCCCAUU
M-058494-01	D-058494-03	Wrn	22427	NM_001122822	GAAAAGUUCCGGUUAUAUA
M-058494-01	D-058494-04	Wrn	22427	NM_001122822	GAUCGACGGUGUCUCUGAA

M-046995-01	D-046995-01	Recql4	79456	NM_058214	CCAAAUCCAUCAACAGUAA
M-046995-01	D-046995-02	Recql4	79456	NM_058214	UGGCAUAGCUGGCGAGUUU
M-046995-01	D-046995-03	Recql4	79456	NM_058214	CCUAGACCCUGGUUGGUUA
M-046995-01	D-046995-04	Recql4	79456	NM_058214	UAUAUUCGACUCAACAUGA
M-045558-00	D-045558-01	Recql5	170472	NM_130454	GGGCCAACCUCUUCUAUGA
M-045558-00	D-045558-02	Recql5	170472	NM_130454	GCUAAUGUCCGGUUUGUUG
M-045558-00	D-045558-03	Recql5	170472	NM_130454	GGGAACAAACCGUCUGAUA
M-045558-00	D-045558-04	Recql5	170472	NM_130454	GAGGGAGGCCGCAGACAUU
M-041700-01	D-041700-01	Rad51b	19363	NM_009014	GCAAACGGCUUAUGAGUUA
M-041700-01	D-041700-02	Rad51b	19363	NM_009014	GAUACCAGAUUGUUAACUG
M-041700-01	D-041700-03	Rad51b	19363	NM_009014	CAAAUUACGACCCAUCUGA
M-041700-01	D-041700-04	Rad51b	19363	NM_009014	CAUAAUGAUGAGUGUCUUA
M-044655-01	D-044655-01	Rad51c	114714	NM_053269	ACAGAGAGUUCUUUGAAUU
M-044655-01	D-044655-02	Rad51c	114714	NM_053269	GAUAAUAGACGGAAUUGCU
M-044655-01	D-044655-03	Rad51c	114714	NM_053269	CGUACUCGAUUACUAAAUG
M-044655-01	D-044655-04	Rad51c	114714	NM_053269	CAGAAGGAGUCUACGAUAC
M-059082-01	D-059082-01	Rad51d	19364	NM_011235	GCGCAGAUCUCUAUGAGGA
M-059082-01	D-059082-02	Rad51d	19364	NM_011235	CAACGGGUCUGCAGGAGAU
M-059082-01	D-059082-03	Rad51d	19364	NM_011235	GGAGCAGAGCCCAGAAUUA
M-059082-01	D-059082-04	Rad51d	19364	NM_011235	GGAUACAGGUGGUGCGUUC
M-058519-00	D-058519-01	Xrcc2	57434	NM_020570	GAGCAGCCGGUUCUCAUUA
M-058519-00	D-058519-02	Xrcc2	57434	NM_020570	GCAGAAGCUCGUUGAAAGA
M-058519-00	D-058519-03	Xrcc2	57434	NM_020570	UUUAACAGCCCGAUGUAUA
M-058519-00	D-058519-04	Xrcc2	57434	NM_020570	GCGACAACGCAGAGUCUAA
M-045249-00	D-045249-01	Fbxo18	50755	NM_015792	GGAGGGAAAUAAUCAAUGA
M-045249-00	D-045249-02	Fbxo18	50755	NM_015792	AAACGAGACCUCAUCAUUA
M-045249-00	D-045249-03	Fbxo18	50755	NM_015792	GCAGAUCUAUACCUUCCGA
M-045249-00	D-045249-04	Fbxo18	50755	NM_015792	GGACUUUGCAGAGUACAUU
M-060606-01	D-060606-01	Chaf1a	27221	NM_013733	GAGGAUGACUCCAUACUGA
M-060606-01	D-060606-02	Chaf1a	27221	NM_013733	UCACACAGGCUCUCACGUA
M-060606-01	D-060606-03	Chaf1a	27221	NM_013733	GAAAAGAGGGACCAGCAUA
M-060606-01	D-060606-04	Chaf1a	27221	NM_013733	GCACGUGGGAUGUGUAUGG
M-061785-01	D-061785-01	Swsap1	66962	XM_913186	CCACAGCGCGAGAGCAUUG
M-061785-01	D-061785-02	Swsap1	66962	XM_913186	UGCCAUUACUUAAGCGAUA
M-061785-01	D-061785-03	Swsap1	66962	XM_913186	AGACGUCGCUGCUGUUUGC
M-061785-01	D-061785-04	Swsap1	66962	XM_913186	AGACAGGUGCAGAUUCAAA
M-060020-01	D-060020-01	Spidr	224008	NM_146068	GCAAGAUGGUGUUUGGUUA
M-060020-01	D-060020-02	Spidr	224008	NM_146068	CGAAAGAGAUCUGCCAUUU
M-060020-01	D-060020-03	Spidr	224008	NM_146068	UAAGACACUUCCUGCAUUA
M-060020-01	D-060020-04	Spidr	224008	NM_146068	GUUAUUGACUGGGAGGUUA
M-053866-01	D-053866-01	Fan1	330554	XM_976717	AGAGAUUGCCUCCGACUUA
M-053866-01	D-053866-02	Fan1	330554	XM_976717	AUUCAACUCUCGUCAGUAA
M-053866-01	D-053866-04	Fan1	330554	XM_976717	CGUAAAUUAACCUGGAUUA
M-053866-01	D-053866-17	Fan1	330554	XM_976717	CGAAAUUGUCCAGACGAUA
M-061987-01	D-061987-01	Blm	12144	NM_001042527	GACACAAUCUGAAGUACUA
M-061987-01	D-061987-02	Blm	12144	NM_001042527	CUAAAUCUAUGGAGGGUUA
M-061987-01	D-061987-03	Blm	12144	NM_001042527	CCUAUGAUAUCGAUAACUU
M-061987-01	D-061987-04	Blm	12144	NM_001042527	ACACCUGCGUUAAGUGAUA
M-064146-01	D-064146-01	Usp1	230484	NM_146144	GUUAUGAGCUUAUAUGUAG
M-064146-01	D-064146-02	Usp1	230484	NM_146144	CACAGUGGCAUUACUAUUA
M-064146-01	D-064146-03	Usp1	230484	NM_146144	CUACGACGAUGAAGUAUCA
M-064146-01	D-064146-04	Usp1	230484	NM_146144	AUUAUGAGCUGUACAACAA
M-059696-00	D-059696-01	Rmi1	74386	NM_001168248	GAAAGGACCCUCUUAAAUA
M-059696-00	D-059696-02	Rmi1	74386	NM_001168248	CAAACCAGCCCACGCAUUU

M-059696-00	D-059696-03	Rmi1	74386	NM_001168248	CUAGAAGGGUUACAGAAAU
M-059696-00	D-059696-04	Rmi1	74386	NM_001168248	GAAUGGAGUAUCAGUCUAU
M-052249-01	D-052249-13	Rmi2	223970	NM_001162932	GGAACUGGAUCCUCGGUUG
M-052249-01	D-052249-14	Rmi2	223970	NM_001162932	CUGCUUACAUGGACGCCUU
M-052249-01	D-052249-15	Rmi2	223970	NM_001162932	CCUGGUUCUUCGAAUGAUA
M-052249-01	D-052249-16	Rmi2	223970	NM_001162932	AGUAUGGCAUGGAUGUUAA
M-041565-01	D-041565-02	Slx1b	75764	NM_029420	CGACCUGACUUCCGCCAUG
M-041565-01	D-041565-03	Slx1b	75764	NM_029420	ACACAUGCCCAUUGCCUUU
M-041565-01	D-041565-04	Slx1b	75764	NM_029420	UCGCAAGAAAGGUGGAGCA
M-041565-01	D-041565-17	Slx1b	75764	NM_029420	GCGCACAUGCUUCGAGUUC
M-049483-00	D-049483-01	Smc1a	24061	NM_019710	GUACAAGGGUCGACAGAUU
M-049483-00	D-049483-02	Smc1a	24061	NM_019710	GAAAGGAGGCCAAACAAGA
M-049483-00	D-049483-03	Smc1a	24061	NM_019710	GCAGGCAUUUGAACAGAUA
M-049483-00	D-049483-04	Smc1a	24061	NM_019710	GAAAUUGGUGUGCGUAACA
M-055713-02-0005	D-055713-14	Rbbp8	225182	NM_001081223	CCUAGACACUGGCGUGAAA
M-055713-02-0005	D-055713-15	Rbbp8	225182	NM_001081223	GCAUUAACCGGCUACGAAA
M-055713-02-0005	D-055713-16	Rbbp8	225182	NM_001081223	AUAUUGAGGUAGUUCGGAA
M-055713-02-0005	D-055713-17	Rbbp8	225182	NM_001081223	AGAUAUGUUUGAUCGGACA
M-040827-01-005	D-040827-01	Msh2	17685	NM_008628	GCAAACAGAUUAAAUUGGA
M-040827-01-005	D-040827-02	Msh2	17685	NM_008628	GAGGAGAGGUUAAAUUUAG
M-040827-01-005	D-040827-03	Msh2	17685	NM_008628	GAAGUUGCAUUUAUUCCAA
M-040827-01-005	D-040827-04	Msh2	17685	NM_008628	GUGCACAGUUUGGAUAUUA

Supplementary Table S2: Oligonucleotide list.

Name	Purpose	Sequence (5' --> 3')
1kb	sgRNA	TGTCGAGCCCGACGCGCGTG
mActinRTPCRP1	primer (qPCR)	GGCTGTATTCCCCTCCATCG
mActinRTPCRP2	primer (qPCR)	CCAGTTGGTAACAATGCCATGT
Top3aRTPCRUP	primer (qPCR)	TGGCTCATGACTTCCAGATG
Top3aRTPCRDN	primer (qPCR)	ATTGGGTTTTACAGCCTTGC
FancaRTPCRUP	primer (qPCR)	TGTCCCGTGATTCTGACTTC
FancaRTPCRDN	primer (qPCR)	ACTCCTCTCCACGCAAAGTG
Ep400RTPCRUP	primer (qPCR)	AAGGAAGGAAGGCTTGTGGT
Ep400RTPCRDN	primer (qPCR)	TCTTCCCTCTTTCCTCACGA
Msh6RTPCRUP	primer (qPCR)	AAGAAGCTGCCAGACCTTGA
Msh6RTPCRDN	primer (qPCR)	GCTTGAGGGTTTGGACGTA
Mlh1RTPCRUP	primer (qPCR)	GGTGGCTTCCTCATCCACTA
Mlh1RTPCRDN	primer (qPCR)	AGAGCAAGCATCTCCTCGTC
Pms2RTPCRUP	primer (qPCR)	ATAACGTGAGCTCCCCAGAA
Pms2RTPCRDN	primer (qPCR)	GAGGACCAGGCAATCTTTGA
Pms1RTPCRUP	primer (qPCR)	ATGGGCAACATGGAATCTGT
Pms1RTPCRDN	primer (qPCR)	TGGGATACAAGCGGGTAGAC
Smarcal1RTPCRUP	primer (qPCR)	AAATCCCATGTGTCGTCGAG
Smarcal1RTPCRDN	primer (qPCR)	GGCTGTGATGGACAACAGTG
FancmRTPCRUP3	primer (qPCR)	CGAATCCTTTTCAGCTCTGG
FancmRTPCRDN3	primer (qPCR)	AGGGGAGCTGTTAGCCATCT
Chaf1bRTPCRUP	primer (qPCR)	CTCCAATCTTGCTCGACACA
Chaf1bRTPCRDN	primer (qPCR)	CCCTCAGGGTCTTTACCACA
Zswim7RTPCRUP	primer (qPCR)	CAGGTGCTAGGGAGTTCTGG
Zswim7RTPCRDN	primer (qPCR)	GCCGCACCTTTTATCCTTCT
Ercc4RTPCRUP	primer (qPCR)	TGGAACAACACAAGCCTGAA
Ercc4RTPCRDN	primer (qPCR)	GCCACAAAGGGTCCAAGTAA
Topbp1RTPCRUP	primer (qPCR)	CACCTTGGAGCAAGTGTTCA
Topbp1RTPCRDN	primer (qPCR)	GTGCGTTGTCAACCAGAAAA
Ercc1RTPCRUP	primer (qPCR)	CCCTGAAAACAGGAGCAAAG
Ercc1RTPCRDN	primer (qPCR)	CACTTGAACCAGCAGCACAC
Pold3RTPCRUP	primer (qPCR)	GAGTGAGCGAAGCTGTTTCC
Pold3RTPCRDN	primer (qPCR)	CCCGAATTTTCTTTCCGTTT
Usp1RTPCRUP	primer (qPCR)	TGGATTATTTGCCGTTGTGA
Usp1RTPCRDN	primer (qPCR)	GGGCATTTCCACCAACTCTA
BlmRTPCRUP	primer (qPCR)	GGTCCAGAAGGACATCCTCA
BlmRTPCRDN	primer (qPCR)	CAGCCATTGTGTCACATTCC
CtIPRTPCRUP	primer (qPCR)	CCCAGGTACCAGATGAGGAA
CtIPRTPCRDN	primer (qPCR)	TACATGTGTGCCCAAGCAAT
Fancd2RTPCRUP3	primer (qPCR)	TGATGAATTTGCCAACCTGA
Fancd2RTPCRDN3	primer (qPCR)	GGCAGGAGGTTGATGACAAT
Xrcc3RTPCRUP3	primer (qPCR)	CCCTGCTGAGACCACTTAGG
Xrcc3RTPCRDN3	primer (qPCR)	GAAGCTCTCCTTCTGCTGGA
Slx4RTPCRUP	primer (qPCR)	CTTTATGCGAGATGCCCACT
Slx4RTPCRDN4	primer (qPCR)	AGTCTCTGCTCGGCTTTCAC
Rmi1RTPCRUP1	primer (qPCR)	TGGCTTTTGGGGTGTAACTG
Rmi1RTPCRDN1	primer (qPCR)	AGCACCACCCCTTTACATGA
Rmi2RTPCRUP2	primer (qPCR)	CTCCACCCCCTAGAAAAAGC
Rmi2RTPCRDN2	primer (qPCR)	AGCACCCCACGAACACATA
Rad51dRTPCRUP5	primer (qPCR)	TTTCCCCTTAAATGGCGCAG
Rad51dRTPCRDN5	primer (qPCR)	ACATTTGCAGCCACACAGAG

Xrcc2RTPCRUP4	primer (qPCR)	GAATCCGGGACAGAGCTCC
Xrcc2RTPCRDN4	primer (qPCR)	GTTTTCCCCGTTCCTTCTGG
Fbxo18RTPCRUP1	primer (qPCR)	TTTGGACCCTTCTTCAGCCA
Fbxo18RTPCRDN1	primer (qPCR)	CACGGTGCCCAAAATGTACT
Chaf1aRTPCRUP3	primer (qPCR)	CTCCATTGCCTCTCTTCCCA
Chaf1aRTPCRDN3	primer (qPCR)	GCTGGTTGGAGAAGAGGAGT
Swsap1RTPCRUP3	primer (qPCR)	ACTTTGGGATGTGACAACCTC
Swsap1RTPCRDN3	primer (qPCR)	TGCCAGCCCTGATGACAATA
SpidrRTPCRUP4	primer (qPCR)	TCCCCTCACAAATACCAGGC
SpidrRTPCRDN4	primer (qPCR)	CCGCCCCTTAAAAGCTTCTT
Fan1RTPCRUP3	primer (qPCR)	ACGGGAGATGAAAGCCAGAA
Fan1RTPCRDN3	primer (qPCR)	AAGCCCTACATCTGCCCTTC
Pif1RTPCRUP1	primer (qPCR)	TGTTAGCCGTGTCCTTCAGT
Pif1RTPCRDN1	primer (qPCR)	TGCGGATGACCTCAGTGATA
Mus81RTPCRUP4	primer (qPCR)	CAGCAGTTCCAGAAGCCTTG
Mus81RTPCRDN4	primer (qPCR)	CACACGCAGCCTTTGTAACT
Eme1RTPCRUP3	primer (qPCR)	GGGGAAAGCATTGTCACTGG
Eme1RTPCRDN3	primer (qPCR)	CCAGTGCCTTCTCCATGTCT
Phf8RTPCRUP1	primer (qPCR)	TGGCAGTACGAAGAGGATGA
Phf8RTPCRDN1	primer (qPCR)	TCGCCTTTTCCTTTCCCAATC
Msh5RTPCRUP2	primer (qPCR)	AGGAGCACAGAGAGCCAAAG
Msh5RTPCRDN2	primer (qPCR)	AGGGAATAATGGAGGAGAGGAA
Mcm3apRTPCRUP4	primer (qPCR)	AGCATGGTGGGTGACATAGG
Mcm3apRTPCRDN4	primer (qPCR)	AAGGGCACTGTCACTAAGGG
AtripRTPCRUP3	primer (qPCR)	TGCGGGTTGAAGGTAGTCAT
AtripRTPCRDN3	primer (qPCR)	GGTCGTCCGCAGTAAATTCC
Smarcad1RTPCRUP2	primer (qPCR)	TCGGGCCTCTTCAACAGATT
Smarcad1RTPCRDN2	primer (qPCR)	ACAGTGCGTAGGTTCCTTCA
Arid1aRTPCRUP2	primer (qPCR)	CTATCCAAAACAGGCCACCAG
Arid1aRTPCRDN2	primer (qPCR)	ATCCCTGCTGAATTGTTGGC
Setd2RTPCRUP4	primer (qPCR)	TTTAAGAAGAGCTCAGCACCTT
Setd2RTPCRDN4	primer (qPCR)	AGTTCTAGAGGCTCTGTCAGA
H2afzRTPCRUP1	primer (qPCR)	GTACTTGAGTTGGCAGGAAATG
H2afzRTPCRDN1	primer (qPCR)	GATCAGCGATTTGTGGATGTG
Msh3RTPCRUP4	primer (qPCR)	AATTTGGACCCTGCCCTGAA
Msh3RTPCRDN4	primer (qPCR)	CCACACTCCACACACAAAACT
Msh4RTPCRUP1	primer (qPCR)	TGCTTGTGTTGTGGGAAATTCA
Msh4RTPCRDN1	primer (qPCR)	TGTCTGCTCACTACCTTGGA
Mlh3RTPCRUP4	primer (qPCR)	CAACTGTGGCTGTGGATGTC
Mlh3RTPCRDN1	primer (qPCR)	CTTTGGTGAAGCGATAGGGA
Mcm9RTPCRUP4	primer (qPCR)	AGTGATCCGAACGAGTCTGG
Mcm9RTPCRDN4	primer (qPCR)	TGGCCGACTGAAGGTGTAAT
Asf1aRTPCRUP2	primer (qPCR)	GCATCGAGGACCTGTCTGAA
Asf1aRTPCRDN2	primer (qPCR)	GATGAGTCCTGCATTCGGTG
Asf1bRTPCRUP2	primer (qPCR)	CGACCTGGAGTGGAAGATCA
Asf1bRTPCRDN2	primer (qPCR)	TGGTAGGTGCAGGTGATGAG
Brip1RTPCRUP1	primer (qPCR)	AGGGGTTATGAGTCGTCTTGT
Brip1RTPCRDN1	primer (qPCR)	GTCCTTTGAGCACGACTTGA
RecqlRTPCRUP1	primer (qPCR)	AGAATGTCAGCAAGTGTCGC
RecqlRTPCRDN1	primer (qPCR)	CGGGGTCAGCTTTTCATTCA
WrnRTPCRUP3	primer (qPCR)	CAAGGTTCTGCTGGACATGG
WrnRTPCRDN3	primer (qPCR)	GAGGTTTGGCACTGGAAAGG
Slx1bRTPCRUP2	primer (qPCR)	GATGGACCATGCAGCAAGAC
Slx1bRTPCRDN2	primer (qPCR)	ATGATCAGCACCATGTCCCA

Smc1aRTPCRUP1	primer (qPCR)	TTGCAGCTGAACGAAAGGAG
Smc1aRTPCRDN1	primer (qPCR)	TCCATCCGCTTCTTGTCCTT
Msh2RTPCRUP1	primer (qPCR)	TTAATAAATGCAGCCCGGGG
Msh2RTPCRDN1	primer (qPCR)	TGTCCACTGTGCTGAAGTTC
1kbTIDEUP1	TIDE	gctcgtagaaggggaggttg
1kbTIDEDN1	TIDE	atagcagctttgctccttcg
1kbTIDEUP2	TIDE	caggaggccttccatctgt
1kbTIDEDN2	TIDE	GGCCCCATTATTGAAGCATT
16bpTIDEUP1	TIDE	attaagggccagctcattcc
16bpTIDEDN1	TIDE	TGGTGCAGATGAACTTCAGG
9kbTIDEUP1	TIDE	CCATGGGCCAGTAGAATGAC
9kbTIDEDN1	TIDE	TTTGAGAAGGTGGCTGTCCT
9kbTIDEUP2	TIDE	AGCATCCCTAACCTGGAAGC
9kbTIDEDN2	TIDE	AAAGATCTGCTTGCCTCTGC
RMDjunct368UPillumina	primer for strand bias	ACACTCTTTCCCTACACGACGCTCTTCCGATCTCCGGGTCCTTCTTGTGTTTC
RMDjunct368DNillumina	primer for strand bias	GACTGGAGTTCAGACGTGTGCTCTTCCGATCTAACAGCTCCTCGCCCTTG
Mlh1e11sgAUP	sgRNA	caccgCATTGACGTCCACGTTCTGA
Mlh1e11sgBUP	sgRNA	caccgCGAAGTTCACTTTCTGCACG

Supplementary Table S3: Shown is one-way ANOVA results comparing top strand retention for each mismatch to one another for each strand polarity sample. Purple numbers indicate p-value ≤0.05, orange number indicates p-values ≥0.05.

Comparison	WT 16bp	WT 1kb	*Mlh1*$^{-/-}$ 16bp	*Mlh1*$^{-/-}$ 1 kb	*Msh2*$^{-/-}$ 16bp	*Msh2*$^{-/-}$ 1 kb	siTop3α 16bp
base 1 vs. base 2	0.6677	0.0165	0.6781	0.0038	0.5654	0.9047	0.7125
base 1 vs. base 3	0.2416	<0.0001	0.1308	<0.0001	0.0658	0.0762	0.4514
base 1 vs. base 4	0.0178	<0.0001	0.0769	<0.0001	0.0202	0.0017	0.0643
base 1 vs. base 5	0.0099	<0.0001	0.025	<0.0001	0.0006	<0.0001	0.019
base 1 vs. base 6	0.0048	<0.0001	0.0052	<0.0001	<0.0001	<0.0001	0.0045
base 1 vs. base 7	0.0003	<0.0001	0.0028	<0.0001	<0.0001	<0.0001	0.001
base 1 vs. base 8	<0.0001	<0.0001	0.0007	<0.0001	<0.0001	<0.0001	0.0002
base 2 vs. base 3	0.9892	0.0196	0.9164	0.0192	0.8413	0.5192	0.9998
base 2 vs. base 4	0.3655	<0.0001	0.7904	0.0013	0.4924	0.0191	0.7047
base 2 vs. base 5	0.2357	<0.0001	0.4483	<0.0001	0.0241	0.0007	0.3446
base 2 vs. base 6	0.1279	<0.0001	0.1323	<0.0001	0.0024	<0.0001	0.1053
base 2 vs. base 7	0.0094	<0.0001	0.076	<0.0001	0.001	<0.0001	0.0247
base 2 vs. base 8	0.0012	<0.0001	0.0185	<0.0001	0.0001	<0.0001	0.0033
base 3 vs. base 4	0.8224	0.0717	>0.9999	0.8406	0.9979	0.5223	0.9136
base 3 vs. base 5	0.6585	0.0104	0.9821	0.0125	0.2875	0.0318	0.5913
base 3 vs. base 6	0.442	0.0022	0.682	0.0001	0.0366	0.0008	0.2232
base 3 vs. base 7	0.0459	<0.0001	0.4978	<0.0001	0.0145	<0.0001	0.0577
base 3 vs. base 8	0.006	<0.0001	0.1716	<0.0001	0.0019	<0.0001	0.008
base 4 vs. base 5	>0.9999	0.9662	0.9984	0.1702	0.6202	0.6753	0.9975
base 4 vs. base 6	0.9967	0.6249	0.8403	0.0015	0.115	0.0358	0.8521
base 4 vs. base 7	0.4766	0.0002	0.6743	<0.0001	0.0482	0.0021	0.4194
base 4 vs. base 8	0.0951	<0.0001	0.2751	<0.0001	0.0064	<0.0001	0.0821
base 5 vs. base 6	>0.9999	0.9908	0.9898	0.2549	0.9238	0.5583	0.994
base 5 vs. base 7	0.6535	0.0013	0.9415	0.0048	0.7061	0.0582	0.7855
base 5 vs. base 8	0.1603	<0.0001	0.5866	<0.0001	0.187	0.0013	0.2424
base 6 vs. base 7	0.8509	0.0061	>0.9999	0.4261	0.9996	0.8158	0.9919
base 6 vs. base 8	0.288	0.0001	0.9568	0.0002	0.7834	0.0544	0.6229
base 7 vs. base 8	0.9583	0.4345	0.9938	0.0149	0.959	0.5371	0.9626

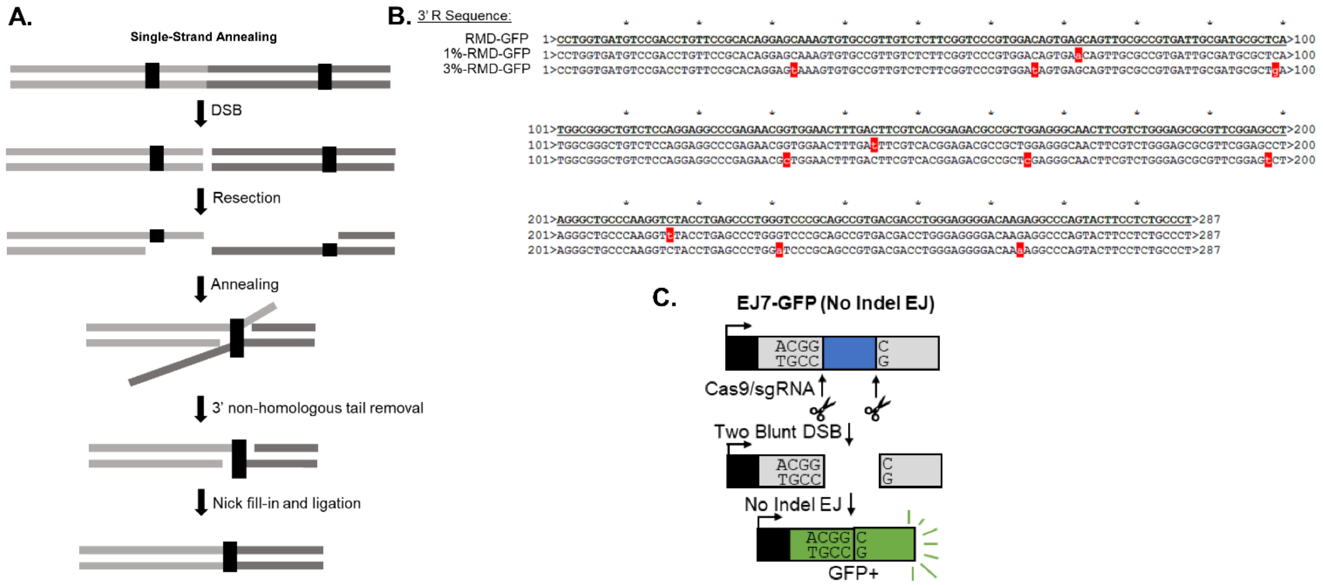

Supplemental Figure S1: Illustrations of SSA model, RMD reporter repeat sequence alignments, and an NHEJ reporter (EJ7-GFP). (A) Shown is a diagram of RMDs via the SSA model. Black boxes represent repeat elements. Following a DNA double strand break (DSB) between the repeats, first step of SSA is 5' to 3' end-resection. The repeats then anneal, forming 3' single-stranded nonhomologous tails which are then cleaved so the resulting DNA nicks can be filled-in and ligated, resulting in an RMD. **(B)** Shown is the sequence of the 3' repeat for each of the three RMD reporters. The top sequence is that of RMD-GFP, the middle 1%RMD-GFP, and the bottom 3%RMD-GFP. The location of each of the mismatches for 1%RMD-GFP and 3%RMD-GFP relative to RMD-GFP are shown in red. Adapted from Mendez-Dorantes et al. 2018 *Genes & Development* 32(7-8):524-536. **(C)** Shown is a diagram for the EJ7-GFP reporter for NHEJ. A GFP cassette is disrupted by a non-homologous insert. Expression of Cas9 and two sgRNAs induce two tandem blunt DSBs that excises the non-homologous insert. NHEJ of the distal blunt DSB ends restores GFP, leading to GFP+ cells. Adapted from Cisneros-Aguirre et al. 2022 *Nature Communications* 13(1):3662.

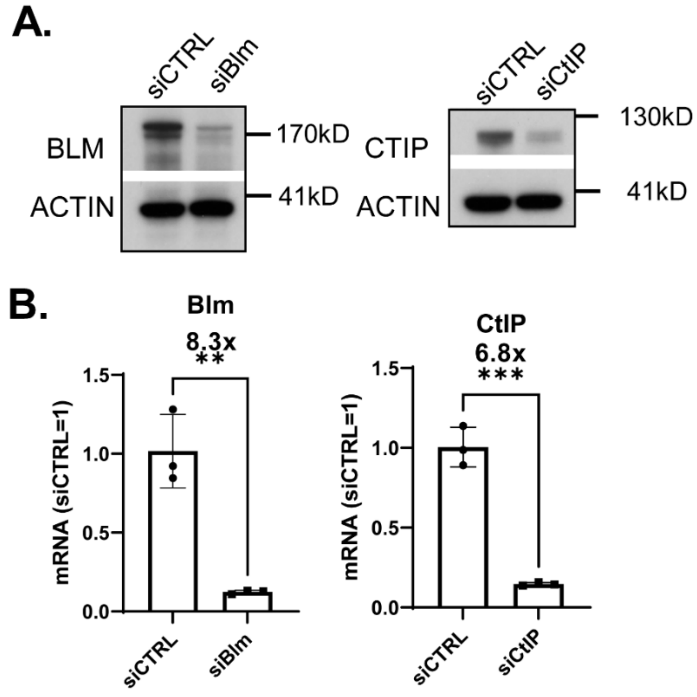

Supplemental Figure S2: Knockdown of BLM and CtIP via siRNA. (A) Immunoblotting analysis of BLM, CTIP, and ACTIN in WT mESCs transfected with either siCTRL, siBlm, or siCtIP siRNAs. **(B)** Shown is qRT-PCR analysis of BLM and CtIP in WT mESCs transfected with either siCTRL, siBlm, or siCtIP siRNAs. Shown is the mRNA abundance of BLM and CtIP based on a threshold cycle (Ct) values from PCR amplification, normalized to ACTIN, relative to siCTRL treated cells (siCTRL = 1). n=3 PCR. **$p \leq 0.005$, ***$p \leq 0.0005$, unpaired t-test. Data are represented as mean values ± SD.

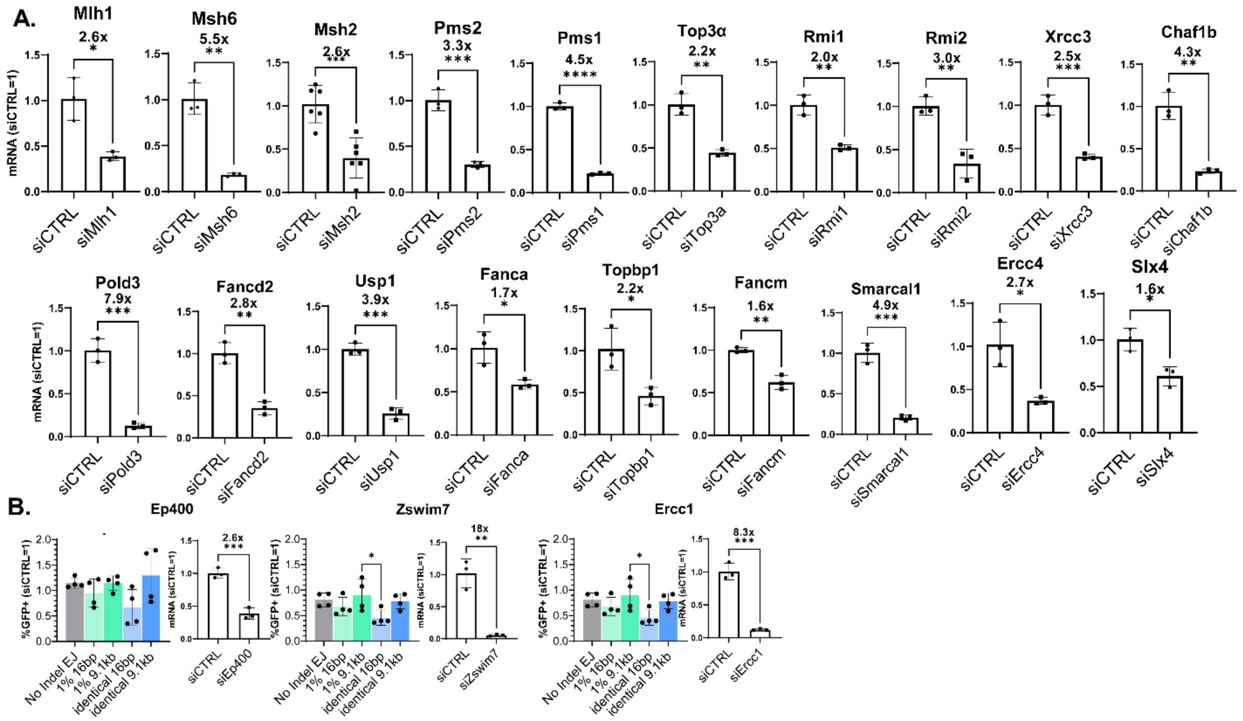

Supplemental Figure S3: qRT-PCR analysis of several siRNAs, and reporter analysis of 3 genes that did not show significant effects on RMDs vs. NHEJ. (A) qRT-PCR analysis of siRNAs targeting the 19 genes described in Figure 3. From siRNA treatment in WT mESCs, shown is the mRNA abundance of each gene based on a threshold cycle (Ct) values from PCR amplification, normalized to actin, relative to siCTRL treated cells (siCTRL = 1). Fold-knockdown shown above each statistical bar. n=3 PCR. $*p \leq 0.05$, $**p \leq 0.005$, $***p \leq 0.0005$, $****p < 0.0001$, Statistics unpaired t-test. **(B)** Effects of siRNAs targeting Ep400, Zswim7, and Ercc1 on the four RMD events and NHEJ. Frequencies are normalized to transfection efficiency and parallel siCTRL (=1). n=4. $*p \leq 0.05$, one-way ANOVA using Tukey's multiple comparisons test. Also shown is qRT-PCR analysis of siRNA treatments targeting these genes, as in (A). $**p \leq 0.005$, $***p \leq 0.0005$, Statistics unpaired t-test, n=3 PCR except n=6 for Msh2. Data are represented as mean values ± SD.

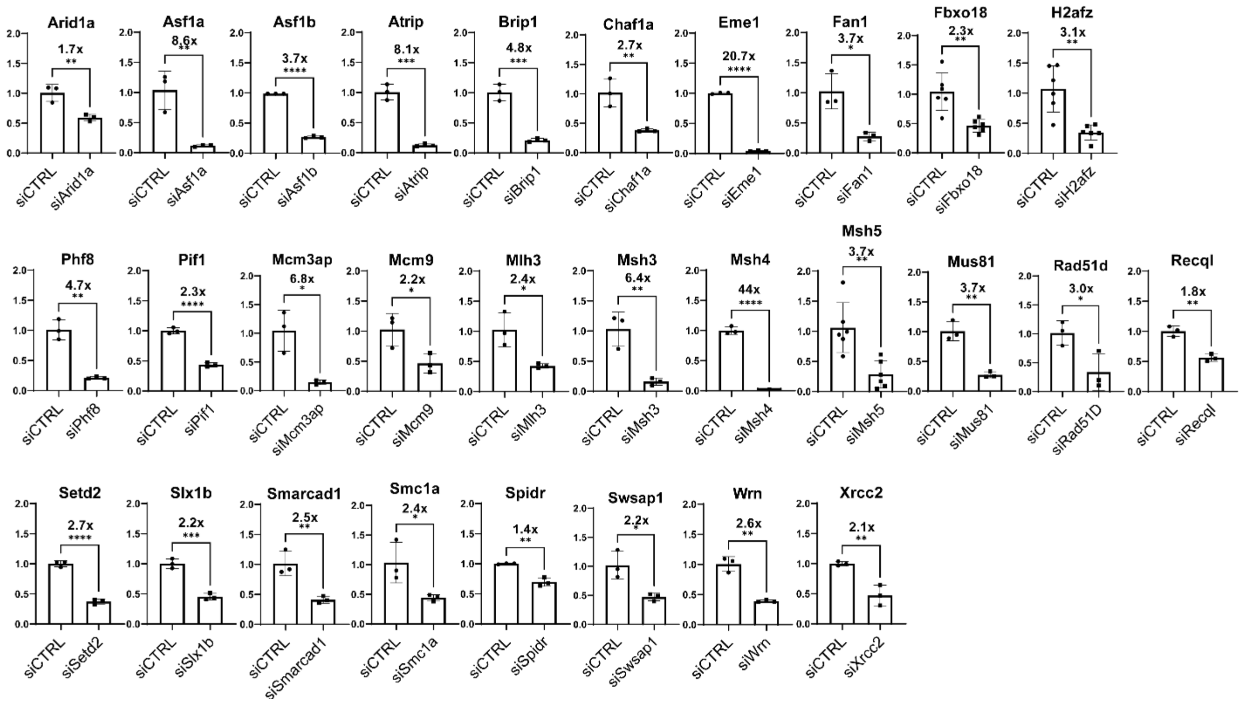

Supplemental Figure S4: qRT-PCR analysis of siRNAs that failed to cause a ≥1.5 fold-effect vs siCTRL on RMDs (see analysis in Figure 2). Shown is qRT-PCR analysis of siRNAs targeting 29 genes with evidence of knockdown, whereas we failed to detect knockdown for the remaining 4 genes analyzed in Figure 2 (Rad51b, Rad51c, Recql4, and Recql5, data not shown). Specifically, following siRNA treatment in WT mESCs, shown is the mRNA abundance of each target gene based on a threshold cycle (Ct) values from PCR amplification, normalized to ACTIN, relative to siCTRL treated cells (siCTRL = 1). Fold-knockdown shown above each statistical bar. n=3 PCR, except n = 6 for Fbxo18, H2afz, and Msh5. *p ≤ 0.05, **p ≤ 0.005, ***p ≤ 0.0005, ****p < 0.0001, Statistics unpaired t-test.

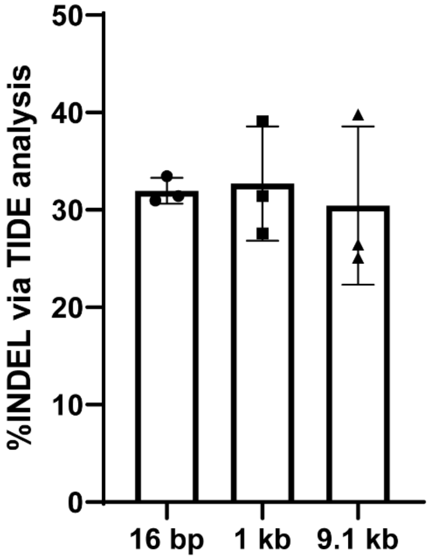

Supplemental Figure S5: Frequency of indels with the sgRNAs targeting the 16 bp, 1 kbp, and 9.1 kbp DSBs. Shown is Tracking of Indels by DEcomposition (TIDE) analysis of samples following single DSB by sgRNAs targeting the 16 bp, 1 kbp, or 9.1 kbp DSB sites. n=3. Data are represented as mean values ± SD.

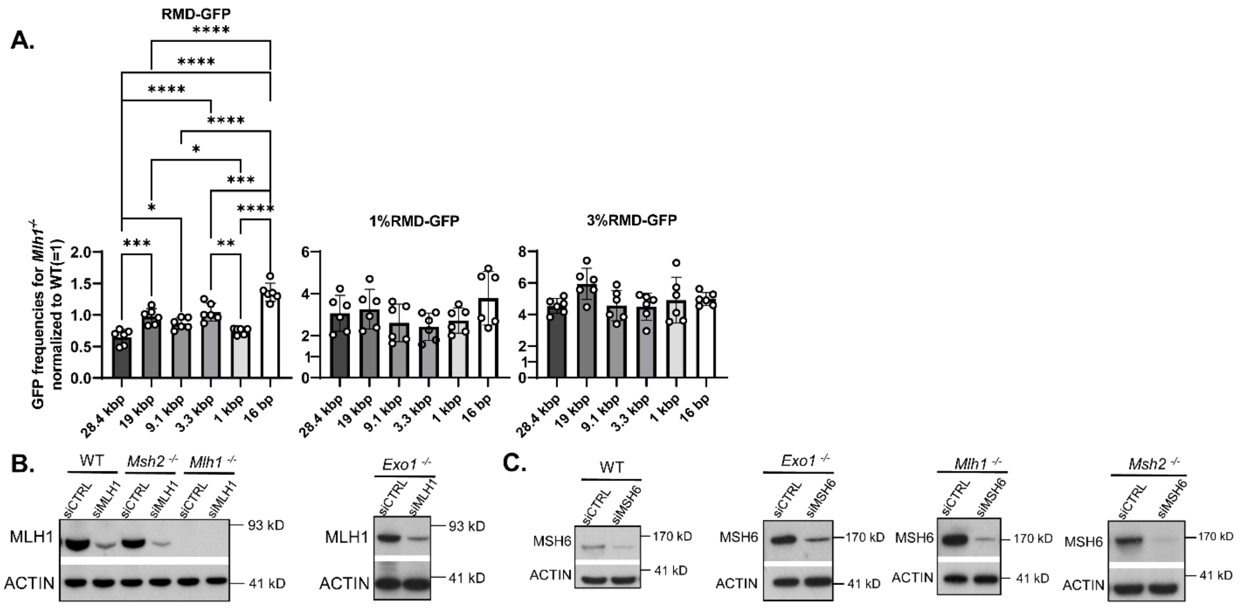

Supplemental Figure S6: MLH1 suppresses RMDs independent of DSB/repeat distance, and validation of MLH1 and MSH6 siRNAs. (A) The RMD frequencies for *Mlh1*$^{-/-}$ shown in Figure 4C were normalized to WT (=1), and grouped by sequence divergence between the repeats to enable comparisons across DSB/repeat distances. n=6. *p ≤ 0.05, **p ≤ 0.005, ***p ≤ 0.0005, ****p < 0.0001, one-way ANOVA with Tukey's multiple comparisons test. Data are represented as mean values ± SD. **(B)** Immunoblotting analysis of MLH1 and ACTIN in WT, *Msh2*$^{-/-}$, *Mlh1*$^{-/-}$, and *Exo1*$^{-/-}$ mESCs that were treated with siMlh1 and siCTRL. **(C)** Immunoblotting analysis of MSH6 and ACTIN in WT, *Msh2*$^{-/-}$, *Mlh1*$^{-/-}$, and *Exo1*$^{-/-}$ mESCs that were treated with siMsh6 and siCTRL.

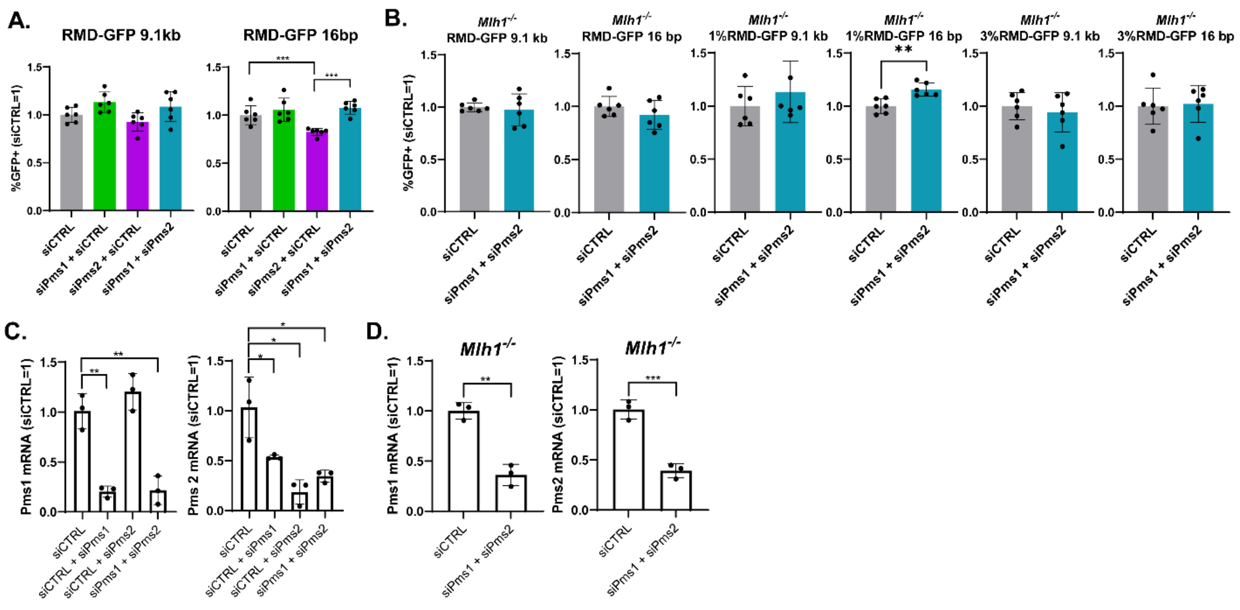

Supplemental Figure S7: Suppression of RMDs via PMS2 and PMS1 is MLH1-dependent. **(A)** Shown is the effects of knockdown of PMS2 and PMS1 individually, and in combination, on RMD events between identical repeats with the 9.1 kbp and 16 bp DSB/repeat distances. Frequencies are normalized to transfection efficiency and parallel siCTRL (=1). n = 6. ***p ≤ 0.0005, siCTRL vs. each set of siRNAs, and the combination siRNA treatment (siPms2 + siPms1) vs. the individual gene siRNAs, using unpaired t-tests with Holm-Sidak correction. **(B)** Shown is the effect of treatment of siRNAs targeting both PMS2 and PMS1 (siPms1 + siPms2) in *Mlh1*$^{-/-}$ mESCs on the three RMD reporters, each tested with the 9.1 kbp and 16 bp DSB/repeat distances. Frequencies are normalized to transfection efficiency and parallel siCTRL (=1). n=6. **p ≤ 0.005, unpaired t-test. **(C)** Shown is qRT-PCR analysis of PMS2 and PMS1 in WT mESCs transfected with either siCTRL, siPms2, siPms1, or both siPms2 + siPms1. Shown is the mRNA abundance of Pms1 and Pms2, based on a threshold cycle (Ct) values from PCR amplification, normalized to actin, relative to siCtrl treated cells (siCTRL = 1). n=3 PCR. *p ≤ 0.05, **p ≤ 0.005, unpaired t-test. **(D)** Shown is qRT-PCR analysis of PMS2 and PMS1 in *Mlh1*$^{-/-}$ mESCs upon transfection with siRNAs targeting PMS2 and PMS1 in combination. Analysis as in (C). Notably, while siRNA targeting PMS2 only affected PMS2 RNA levels, siRNA targeting PMS1 caused a modest but consistent decrease in PMS2 RNA. Although, the combination of siRNA targeting PMS1 and PMS2 showed a similar knockdown of PMS2 RNA as the siRNAs targeting PMS2 alone. In contrast, as described in the Results, the combination siRNA targeting of PMS1 and PMS2 caused the greatest increase in RMDs. n=3. **p ≤ 0.005, ***p ≤ 0.0005, Statistics are unpaired t-test. Data are represented as mean values ± SD.

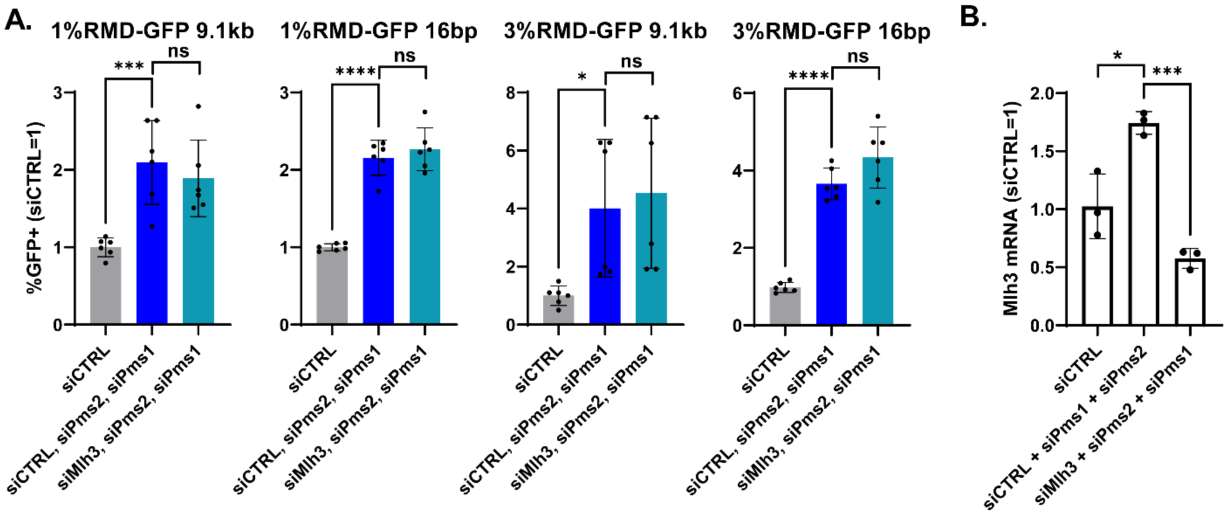

Supplemental Figure S8: RMD frequencies are not obviously affected by siRNAs targeting knockdown of Mlh3. (A) Shown are the effects of combined loss of PMS2 and PMS1, as well as combined loss of PMS2, PMS1, and MLHL3 on RMD events between divergent repeats with the 9.1 kbp and 16 bp DSB/repeat distances. Frequencies are normalized to transfection efficiency and parallel siCTRL (=1). n = 6. ns = not significant, *$p \leq 0.05$, ***$p \leq 0.0005$, ***$p \leq 0.00001$, siCTRL vs. siPms2 + siPms1, and siPms2 + siPms1 vs. siPms2 + siPms1 + siMlh3, using unpaired t-tests. **(B)** Shown is qRT-PCR analysis of Mlh3 in WT mESCs transfected with either siCTRL, the combination of siPms2 and siPms1, or the combination of siPms2, siPms1, and siMlh3. Shown is the mRNA abundance of Mlh3, based on a threshold cycle (Ct) values from PCR amplification, normalized to ACTIN, relative to siCtrl treated cells (siCTRL = 1). Notably, we found Mlh3 RNA was modestly higher in the cells with knockdown of Pms2 and Pms1 (compared to non-targeting siCTRL). In any case, the Mlh3 siRNA caused knockdown of Mlh3 RNA in these cells (i.e., in cells with combined siRNA knockdown of Pms1, Pms2, and Mlh3). n=3 PCR. *$p \leq 0.05$, ***$p \leq 0.0005$, unpaired t-test. Data are represented as mean values ± SD.

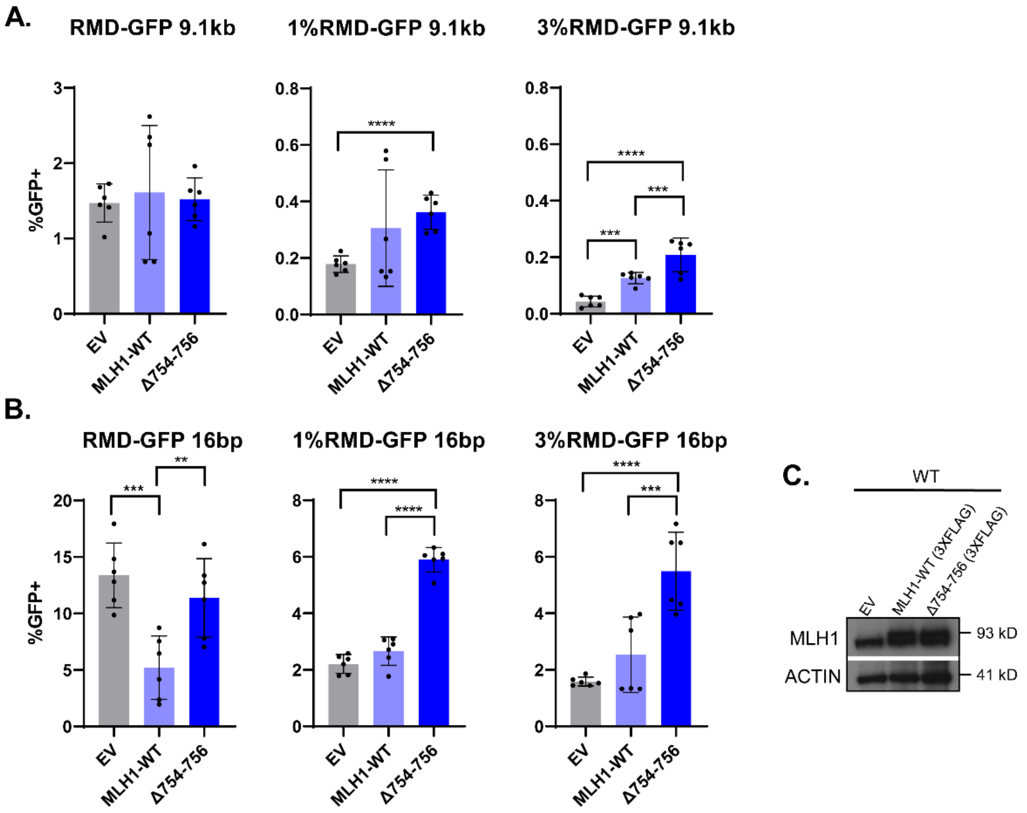

Supplemental Figure S9: Expression of MLH1-Δ754-756 in WT cells causes an increase in the frequency of divergent RMDs. (A) Shown are RMD frequencies for 3 RMD events: RMD-GFP, 1%RMD-GFP, and 3%RMD-GFP at the 9.1 kb DSB/repeat distance. WT cells were transfected with EV, MLH1-WT, or MLH1-Δ754-756. Frequencies are normalized to transfection efficiency. n=6. ***p ≤ 0.0005, ****p < 0.0001, unpaired *t*-tests with Holm-Sidak correction. **(B)** Shown are the 3 RMD events as in (A) but at the 16 bp DSB/repeat distance. As in (A), WT cells were transfected with EV, MLH1-WT, or MLH1-Δ754-756. Frequencies are normalized to transfection efficiency. n=6. **p ≤ 0.005, ***p ≤ 0.0005, ****p < 0.0001, unpaired *t*-tests with Holm-Sidak correction. **(C)** Immunoblotting analysis of MLH1 and ACTIN in WT mESCs transfected with EV, MLH1-WT, or MLH1-Δ746-756.

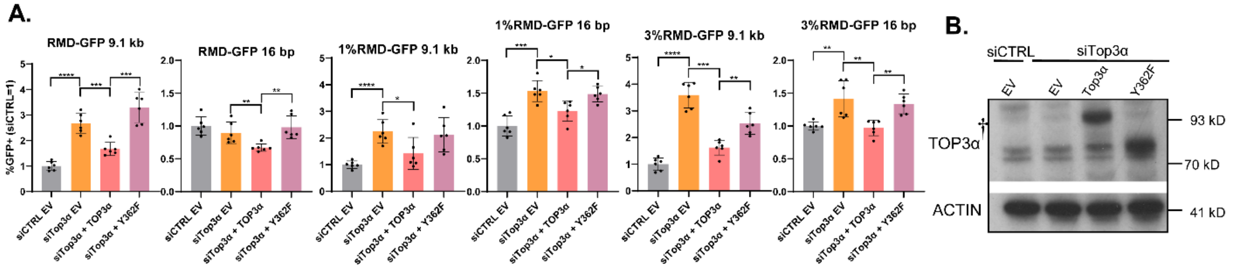

Supplemental Figure S10: Effect of TOP3α Y362F on RMD frequency. (A) Shown are effects of expressing TOP3α WT and Y362F in cells treated with siTop3 for 6 RMD events: RMD-GFP, 1%RMD-GFP, and 3%RMD-GFP, each with the 9.1 kbp and 16 bp DSB/repeat distances. Frequencies are normalized to transfection efficiency and parallel siCTRL (=1). n=6. *$p \leq 0.05$, **$p \leq 0.005$, ***$p \leq 0.0005$, ****$p < 0.0001$, siCTRL EV vs. siTop3a EV unpaired t-test, and siTop3a EV vs. siTop3a TOP3α and siTop3a TOP3α vs. siTop3a TOP3α-Y362F unpaired t-tests with Holm-Sidak correction. Data are represented as mean values ± SD. **(B)** Immunoblotting analysis of TOP3A and ACTIN in WT mESCs transfected with either siCtrl EV, or siTop3a with EV, WT TOP3α, or Y362F. Same blot as in Figure 3D but with un-cropping the Y362F lane. The † symbol notes that endogenous mouse Top3α is not readily detected by this antibody raised against the human protein.

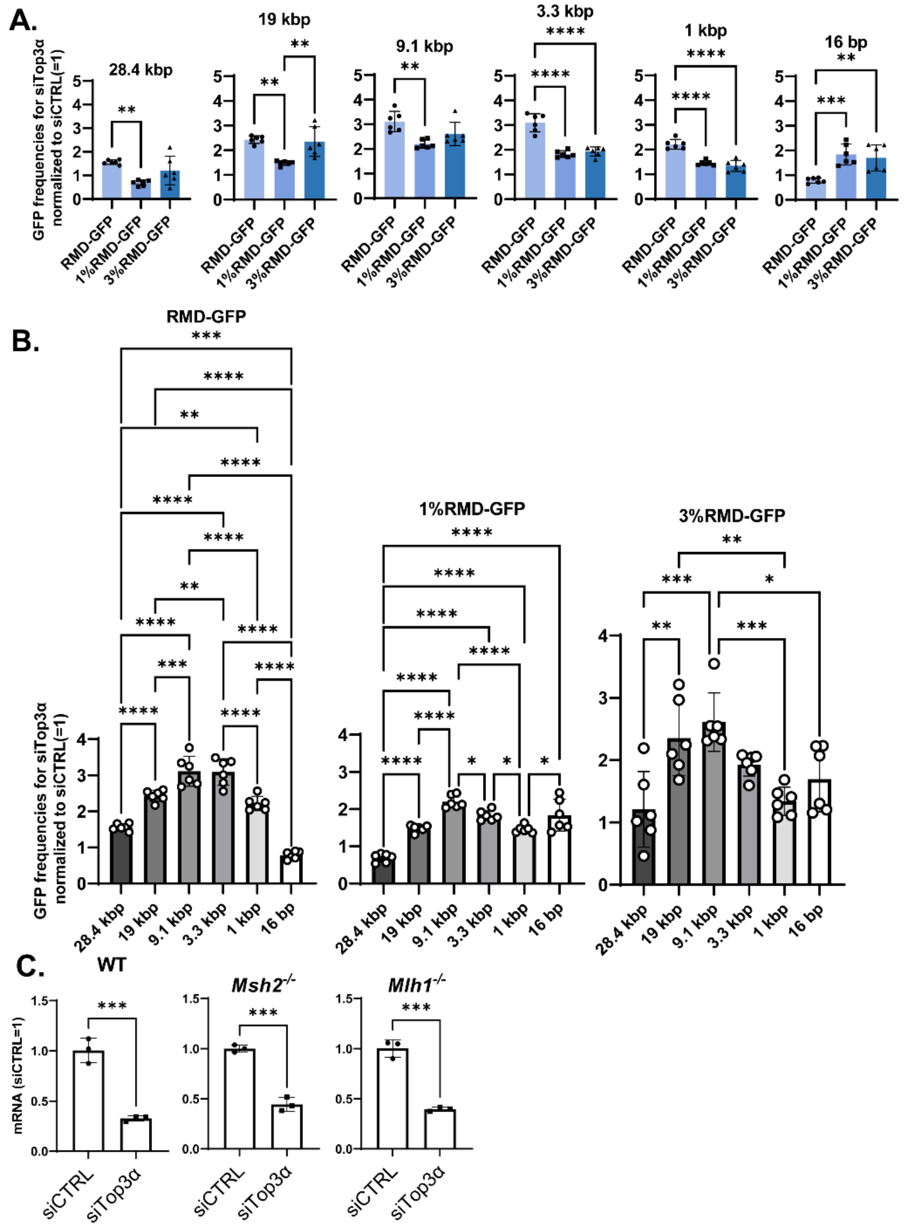

Supplemental Figure S11: **Influence of sequence divergence and DSB/repeat distance on the relative fold effect of TOP3α on RMDs, and assessment of TOP3α knockdown in various contexts. (A)** RMD frequencies from siTop3α treated cells shown in Figure 6A were normalized to siCTRL (=1), and grouped by sequence divergence to enable comparisons of effects of DSB/repeat distance. n=6. **$p \leq 0.005$, ***$p \leq 0.0005$, ****$p < 0.0001$, one-way ANOVA with Tukey's multiple comparisons test. **(B)** Analysis as in (A) except grouped by DSB/repeat distances to enable comparisons with varying degrees of sequence divergence. n=6. *$p \leq 0.05$, **$p \leq 0.005$, ***$p \leq 0.0005$, ****$p < 0.0001$, one-way ANOVA with Tukey's multiple comparisons test. **(C)** Shown is qRT-PCR analysis of TOP3α in WT, *Mlh1*$^{-/-}$, and *Msh2*$^{-/-}$ mESCs upon transfection with siRNAs targeting TOP3α. Shown is the mRNA abundance of TOP3α based on a threshold cycle (Ct) values from PCR amplification, normalized to actin, relative to siCTRL treated cells (siCTRL = 1). n=3 PCR. ***$p \leq 0.0005$, unpaired t-test.
